# Metatranscriptomic and comparative genomic insights into resuscitation mechanisms during enrichment culturing

**DOI:** 10.1186/s40168-018-0613-2

**Published:** 2018-12-26

**Authors:** Da-Shuai Mu, Qi-Yun Liang, Xiao-Man Wang, De-Chen Lu, Ming-Jing Shi, Guan-Jun Chen, Zong-Jun Du

**Affiliations:** 1 State Key Laboratory of Microbial Technology, Institute of Microbial Technology, Shandong University, Qingdao, 266237 China; 2 College of Marine Science, Shandong University, Weihai, 264209 People’s Republic of China

**Keywords:** Enrichment culture, Mixed culture, Metatranscriptomics, Resuscitation, Uncultured bacteria

## Abstract

**Background:**

The pure culture of prokaryotes remains essential to elucidating the role of these organisms. Scientists have reasoned that hard to cultivate microorganisms might grow in pure culture if provided with the chemical components of their natural environment. However, most microbial species in the biosphere that would otherwise be “culturable” may fail to grow because of their growth state in nature, such as dormancy. That means even if scientist would provide microorganisms with the natural environment, such dormant microorganisms probably still remain in a dormant state.

**Results:**

We constructed an enrichment culture system for high-efficiency isolation of uncultured strains from marine sediment. Degree of enrichment analysis, dormant and active taxa calculation, viable but non-culturable bacteria resuscitation analysis, combined with metatranscriptomic and comparative genomic analyses of the interactions between microbial communications during enrichment culture showed that the so-called enrichment method could culture the “uncultured” not only through enriching the abundance of “uncultured,” but also through the resuscitation mechanism. In addition, the enrichment culture was a complicated mixed culture system, which contains the competition, cooperation, or coordination among bacterial communities, compared with pure cultures.

**Conclusions:**

Considering that cultivation techniques must evolve further—from axenic to mixed cultures—for us to fully understand the microbial world, we should redevelop an understanding of the classic enrichment culture method. Enrichment culture methods can be developed and used to construct a model for analyzing mixed cultures and exploring microbial dark matter. This study provides a new train of thought to mining marine microbial dark matter based on mixed cultures.

**Electronic supplementary material:**

The online version of this article (10.1186/s40168-018-0613-2) contains supplementary material, which is available to authorized users.

## Background

The ocean microbiome was one of the first microbiomes to be studied, but the diversity and distribution of its members are only now becoming familiar [1, 2]. However, key questions remain because most microbial species in the biosphere resist cultivation in the laboratory [3, 4]. These species are often referred to as microbial “dark matter” [5, 6]. Researchers have pointed out that if one could provide the chemical components of natural environment, uncultured microbes might grow under pure culture condition. To provide these components and allow the culturing of important microbial taxa, some cultivation methods such as culture cocktails [7], miniaturized cultures [8], diffusion chambers [9], and nature’s incubator [10] have been developed.

These have been great successes; however, many microbial species in the biosphere that would otherwise be “culturable” may fail to grow because of their growth state in nature, such as dormancy [11], which results in species that are referred to as viable but non-culturable (VBNC) [12]. Sediments can be highly variable in terms of nutrient input and cells might be dormant because of the lack of a major nutrient and/or carbon source even though they naturally occur in these sediments. As a result, nature’s incubator [10] and diffusion chambers [9], in which diffusion provides microorganisms with their natural environment incubated in situ, cannot easily capture dormant microbes. Recent studies have shown that marine environments are a “seed bank,” where dormant bacteria are still present in the marine sediments but at much lower abundance [13]. If one could predict the metabolism of an organism and provide the preferred carbon/nutrient sources, one should be theoretically able to switch a dormant to a non-dormant microbe.

Moreover, Lewis and Epstein reported that some isolates merely grew with mixed-culture [9], thereby demonstrating mixed-culture dependence for some certain isolates [14]. Mixed cultures can provide a simple enough community to research the individual community members [15], to fully understand the communication of microorganisms, [16], and represent strategies to grow unculturable bacteria [14].

In fact, mixed cultures are not a recent idea. *Clostridium pasteurianum* was isolated through mixed culture by Sergei Winogradsky in 1895 [17], which were a type of mixed culture. Enrichment is a long-used and common practice and effectively increases the population of target organisms. By using the enrichment culturing method, several rare microorganisms belonging to infrequently isolated or recently described taxa were isolated in our previous study, including *Bradymonadales* [18], *Marinilabiliales* [19], and *Draconibacteriaceae* [20]. Furthermore, the enrichment culturing method has been helpful for single-cell genomics analysis [21]. However, the many questions that remain regarding enrichment culturing limit its application in the isolation of microbial dark matter. For example, how and why microorganism communities change during the enrichment culturing is unknown, as is, more importantly, whether “yet-to-be cultivable” strains can be isolated due to their enriched abundance. Clarifying these questions might be helpful for precisely culturing microorganisms in mixed cultures for drug discovery or screening novel natural products that may finally end the antibiotics discovery void.

**Fig. 1:**
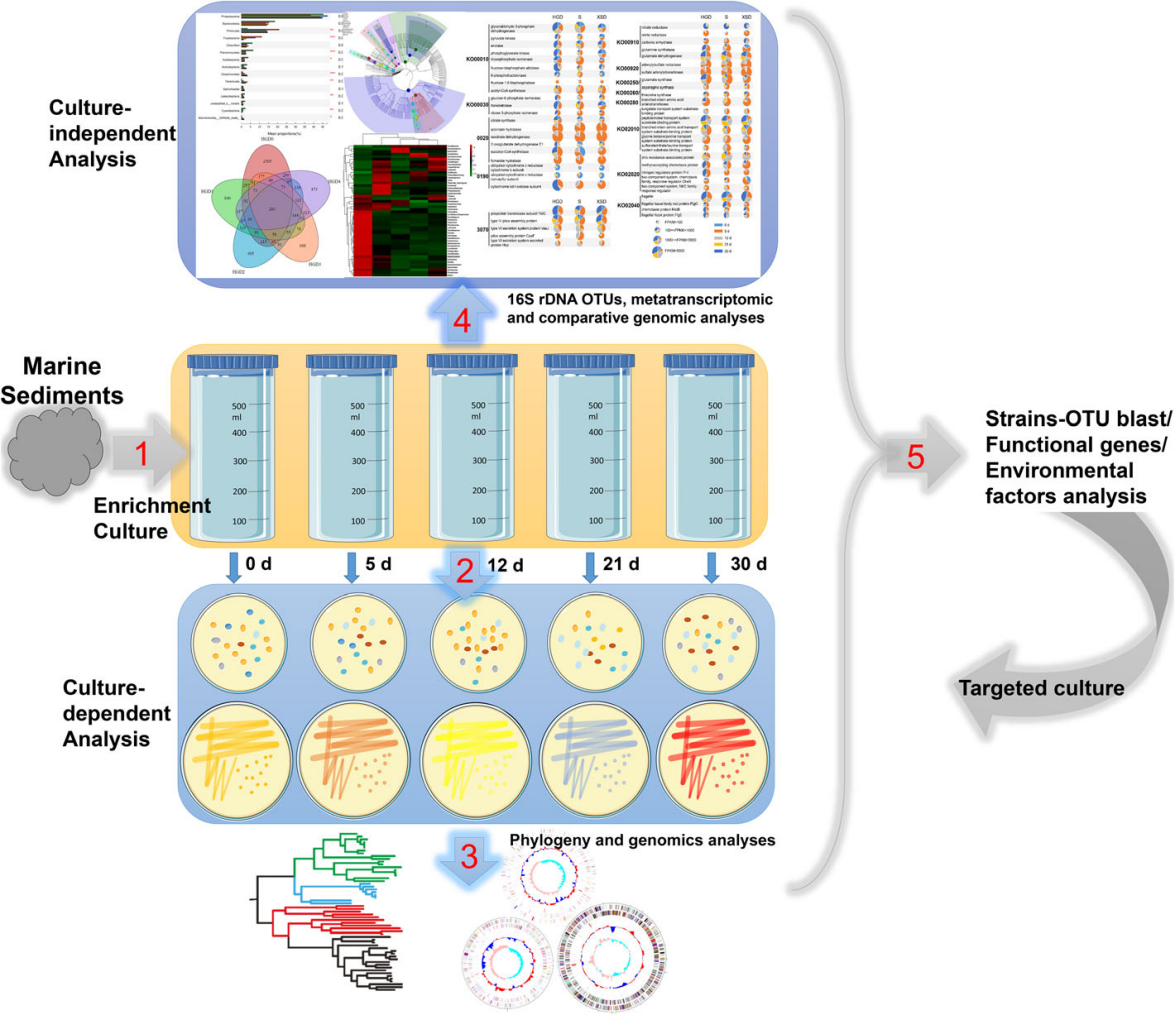
A workflow for culturing, archiving, and characterizing sediment microbiotas. Schematic diagram of the workflow, encompassing bacterial culturing and genomics to isolate and characterize bacterial species from a marine sediment microbiota. The process incorporates the following steps: resuscitation cultures (0–30 days), restreaking, archiving, and phenotyping. First, fresh sediment samples are cultured in the enrichment culture medium for the isolation of bacteria. The anaerobic enrichment cultures are diluted serially diluted, and aliquots of the homogenate are subsequently inoculated on MA to isolate bacteria. Second, isolates are identified by selecting single colonies that are streaked to purity, and full-length 16S rRNA genes are amplified and sequenced. Third, each unique, novel, desired isolate is archived frozen in a culture collection, and a whole-genome sequence is generated. Finally, a metatranscriptomic analysis of the microbiota during each period of resuscitation culturing is conducted and combined with information from environmental factors, comparative genomic analysis, and a local BLAST of cultured strains

Here, we constructed an enrichment culture system for the high-efficiency isolation of uncultured strains of marine sediment bacteria that are not commonly found in 16S rRNA gene clone libraries from marine sediment (Fig. 1). Using this method enabled us to achieve a high isolation rate for novel species. Combining this approach with metatranscriptomic analysis, ecological analysis, and VBNC resuscitation growth studies revealed a resuscitation mechanism during enrichment culture. Meanwhile, metatranscriptomic and comparative genomic analyses of some isolates and the enriched microbes revealed syntrophic partnerships based on vitamin exchange in enrichment cultures. Based on the resuscitation mechanism, our system could be a broad method for mining the marine microbial dark matter.

## Results and discussion

### Culturing the marine sediment microbiotas based on the enrichment method

We developed an enrichment culture method using a low-nutrient medium containing 10 mM sodium pyruvate in the culture (Fig. 1) because low-nutrient media facilitated the cultivation of previously uncultured marine bacterioplankton [11]. Meanwhile, sodium pyruvate was reported to have a resuscitative effect on VBNC *Salmonella* [22] and shortened the lag phase of *Nitrosomonas europaea* during mixed culture stimulation [23].

**Fig. 2:**
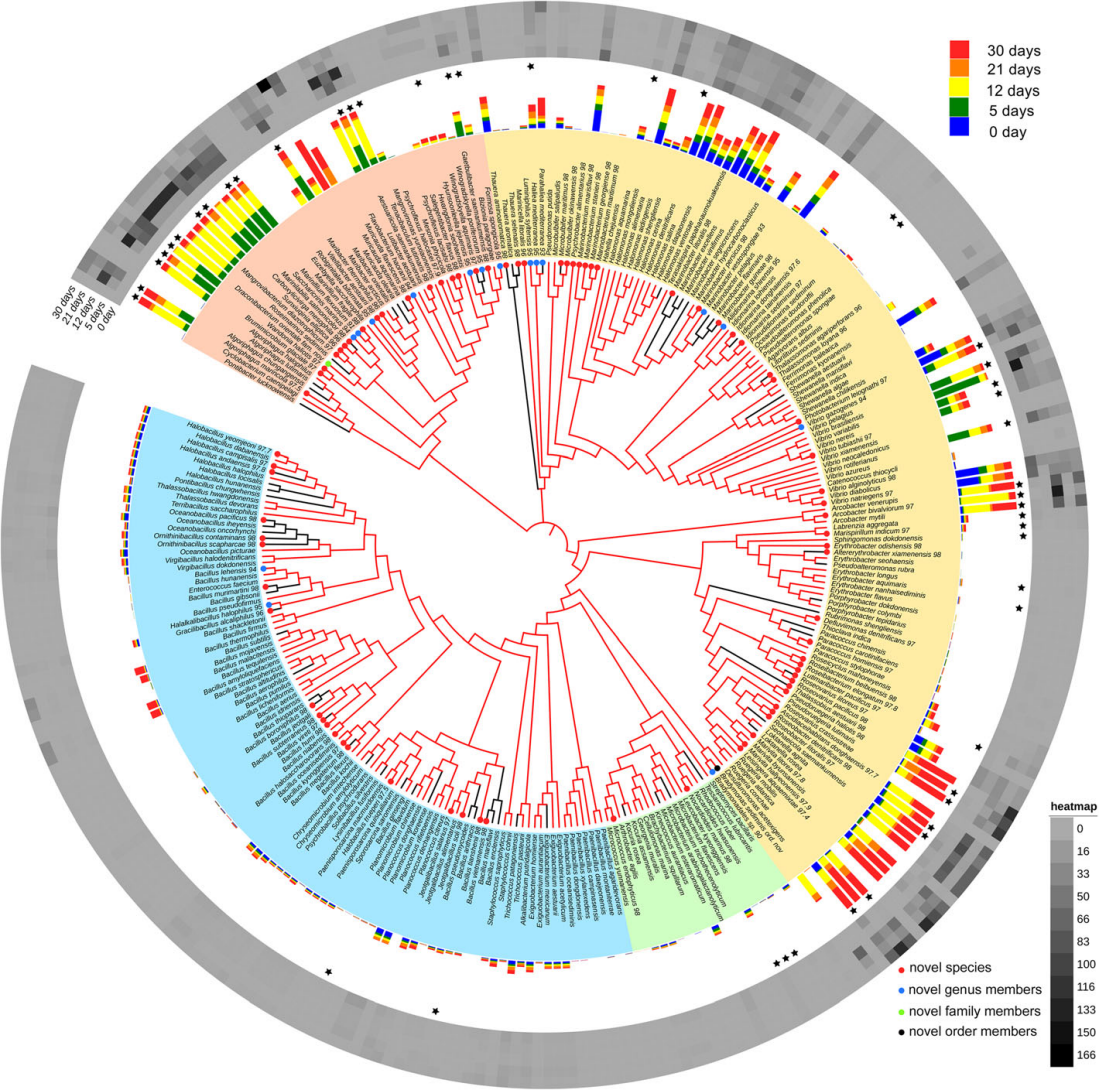
Phylogenetic tree of bacteria cultured from the three marine sediment samples and correlation analysis of cultured bacteria and total communities during enrichment culturing. The phylogenetic tree was constructed from almost full-length 16S rRNA gene sequences (amplified using 27F/1492R). Red tree branches indicate that the species was isolated after enrichment culture, while black tree branches indicate that the species was isolated by directly culture on MA (enrichment culture of 0 days). Novel candidate species (red circle, 16S rRNA gene similarity< 98%), genera (blue circle, similarity< 95%), families (green circle, similarity< 90%), and orders (black circle, similarity< 86%) are indicated by colored dots on the branch. Major phylum names are indicated and marked in different colors. An orange background for species indicates the phylum Bacteroidetes, a yellow background indicates the phylum Proteobacteria, a green background indicates the phylum Actinobacteria, and a blue background indicates the phylum Firmicutes. The bar chart indicates that the mean reads number of 282 cultured species during different periods of enrichment (blue bar indicates 0 days of enrichment, green bar indicates 5 days after enrichment, yellow bar indicates 12 days after enrichment, orange bar indicates 21 days after enrichment, and red bar indicates 30 days after enrichment). The heatmap indicates the mean read abundance of 282 cultured species during different periods of enrichment (color ranging from gray to black represents a read number from 0 to 166 in rarefied sequences). Black star indicates the isolated species were enriched (enrichment ratio > 1) after enrichment culture. In addition, detailed information of each species is shown in Additional file 3: Tables S3, Additional file 4: Table S4, and Additional file 5: Table S5.

For the systematic analysis of this method, three marine sediment samples were separately enrichment cultured from 0 to 30 days. The number of cultured bacteria was highest on the fifth day and then gradually decreased as the culture time was extended (Additional file 1: Table S1). A total of 1251 individual bacterial colonies were selected by using this method, and 16S rRNA gene (almost full-length) analysis showed that the isolates were mainly distributed throughout 282 species of four phyla, Firmicutes, Proteobacteria, Bacteroidetes, and Actinobacteria, and contained 97 candidate novel species (including 1 candidate novel order, 1 candidate novel family, 16 candidate novel genera, and 79 candidate novel species) (Fig. 2, Additional file 2: Table S2, Additional file 3: Table S3, and Additional file 4: Table S4).

The quantities of specific isolates differed at various stages in the enrichment culture, and 2- to 4-fold more different species could be obtained from the same sediment sample after enrichment culturing (Additional file 5: Table S5). The culturable bacterial species isolated from different marine sediment samples were different, and only approximately 20% of the species were isolated in two sediment samples (Additional file 5: Table S5). However, the different samples in an enrichment culture exhibited similarities: (a) Proteobacteria are most abundant in the samples at various times during enrichment culturing. (b) The number of culturable Bacteroidetes isolates increased after enrichment culturing. (c) Some novel species could be isolated only after enrichment culturing, including some members of *Marinilabiliales*, ε-*Proteobacteria*, and δ-*Proteobacteria*. These results suggested that the method can change the flora of cultivable microbial communities and can be very effective in the separation of marine sediment bacteria.

### Composition and diversity of microbial communities in enrichment culture

To explore the compositional changes of microbial communities in different enrichment culture stages, the 16S rRNA gene PCR products of the V4-V5 region were sequenced. The barcoded high-throughput sequencing generated 914,059 quality sequences from 15 samples (Additional file 6: Table S6), with an average of 60,937 sequences per treatment. The number of OTUs detected in each sample ranged from 1285 to 2982 (Additional file 6: Table S6). Bacteria were determined to be more relatively abundant in samples from day 0 than later samples by analysis of the ACE (abundance-based coverage estimator), Chao1 estimator, and Shannon and Simpson indexes (Additional file 6: Table S6). The rarefaction curves, which were based on OTU numbers, almost reached the asymptote for different samples (Additional file 7: Figure S1a); the Shannon diversity index curves also reached plateau levels (Additional file 7: Figure S1b). These results suggested that the vast majority of prokaryotes were well represented in these reservoirs. However, new unique OTUs appeared after the enrichment treatment, and the number of novel OTUs was 16.8% greater after the enrichment cultivation than in the original sediment samples (Additional file 7: Figure S1c). Thus, sequencing analyses would miss some low-abundance sequences of rare strains, i.e., the real microbial dark matter. However, enrichment culture could change the abundance of some OTUs, allowing some low-abundant OTUs to reach the detection threshold. Principal components analysis (PCoA) based on weighted UniFrac metrics also showed that the OTUs found in each enrichment treatment were different in their abundance from each other and that the different sediment samples showed different OTUs under the same enrichment treatment (Additional file 7: Figure S1d).

Of the classifiable sequences, 53 phyla were identified, with Proteobacteria, Fusobacteria, Bacteroidetes, Firmicutes, and Chloroflexi representing the most dominant lineages in the samples receiving the 5-stage treatment (Additional file 7: Figure S1e). During enrichment cultivation, the abundance of the phyla Fusobacteria and Firmicutes increased significantly, whereas that of phyla Planctomycetes and Acidobacteria decreased (Additional file 7: Figure S1). At the family level, some anaerobes or facultative anaerobes, such as *Marinilabiaceae*, *Fusobacteriaceae*, and *Clostridiaceae*, were significantly enriched under enrichment culturing (Additional file 7: Figure S1f).

These results strongly indicate that the composition and diversity of microbial communities changed with environmental factors and that novel operational taxonomic units (OTUs) appeared after enrichment, suggesting that marine sediment may contain a “microbial seed bank” [13] or a set of rare microbes that can barely survive until the proper nutrients arrive [24, 25].

### Mechanism of culturing the isolates

It is usually considered that enrichment culturing enhances the probability of isolating uncultured microorganisms by increasing their abundance. However, few systemic analyses of the mechanism responsible for culturing isolates during enrichment exist. Whether the “yet-to-be cultivable” strains are cultured due to their abundance enrichment thus remains unclear.

**Fig. 3:**
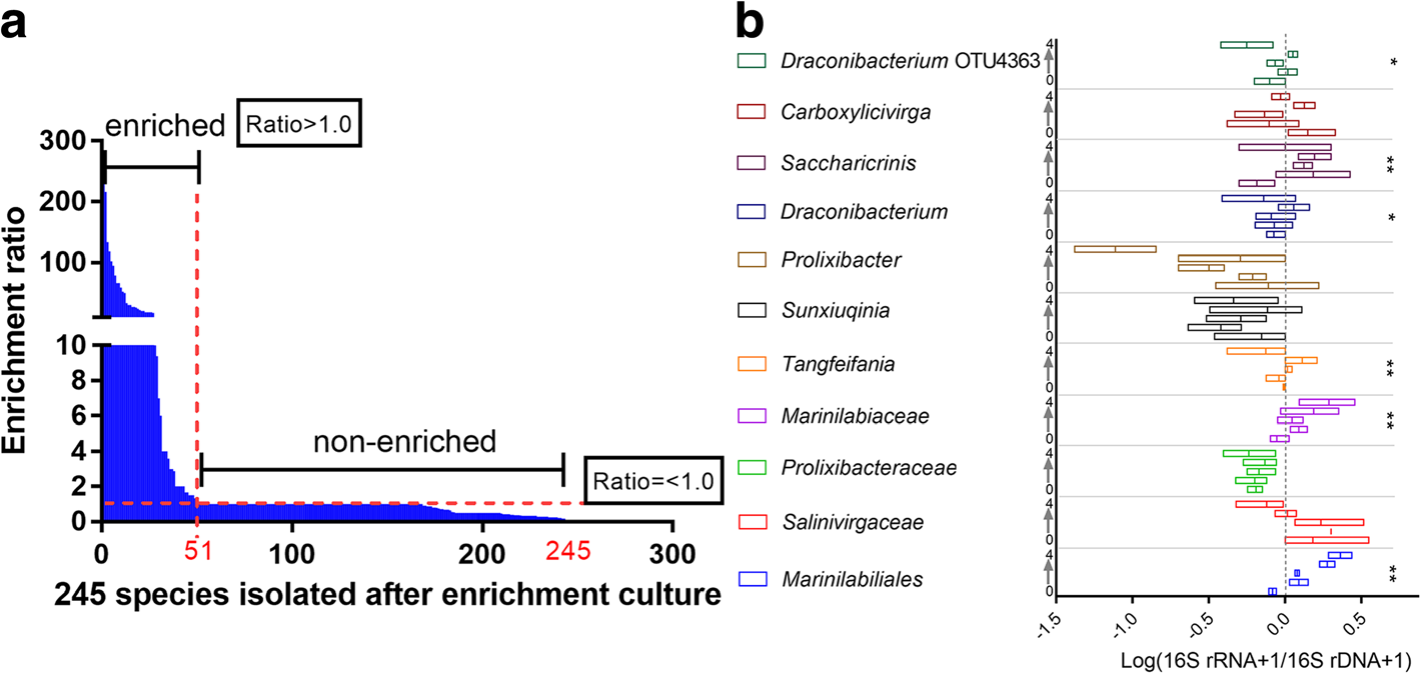
Enrichment ratio of the cultured species and growth states of *Marinilabiliales* during the enrichment culturing. **a** The enrichment ratio or enrichment degree of the cultured species. The enrichment ratio indicates the abundance of a cultured species in the corresponding period of enrichment culturing relative to its original abundance. A ratio > 1 means that the species was enriched when it was isolated. In contrast, a ratio = < 1 means that the species was not enriched when it was isolated. **b** Dormant and active bacteria of *Marinilabiliales* during the enrichment culture. Different levels of taxonomic composition of original sediment and enrichment treatments were assessed according to the log10 16S rRNA:16S rRNA gene ratio for the microbial order *Marinilabiliales*. The dotted line represents a 16S rRNA:16S rRNA gene ratio of 1. Taxa with ratios higher than 1 are considered active, and taxa with ratios lower than 1 are considered dormant. The numbers 0 to 4 (each box from bottom to top in one group) indicate the 0, 5, 12, 21, and 30 days of enrichment culturing. Boxes represent quartiles, and the solid line is the median value (*n* = 3,**p < 0.01, *p < 0.05).

To answer the above questions, we conducted a local BLAST analysis of the 16S rRNA gene of 282 isolated species against 16S rRNA gene libraries of different stages of enrichment. The results showed that 16S rRNA gene sequences (from the MiSeq dataset) that were homologous to those of most of the cultured strains were not detected in the 16S rRNA gene libraries, which suggests that most of the microorganisms isolated by this method were not abundant (bar chart and heatmap analysis in Fig. 2). Furthermore, we analyzed the degree of enrichment of 245 species that were isolated after enrichment culturing (Additional file 5: Table S5). The results showed that approximately 80% of the strain isolations were not due to an enriched abundance in the enrichment culture (Figs. 2 and 3a, Additional file 5: Table S5).

However, the abundances of some isolates were enriched at certain enrichment stages. The species most typical of this enrichment is *Draconibacterium orientale* (belonging to a novel genus and a novel family), which was isolated by this method [20]. In our previous long-term studies, this species was recalcitrant to be isolated by directly culturing on MA medium, except after enrichment culture. In addition, strains of this species were repeatedly isolated in these three sediment samples after enrichment. Local BLAST analysis revealed that the abundance of this bacterium was very low in the original samples (usually undetected) but significantly increased with a prolonged enrichment culturing (to 251 reads) (Fig. 2, Additional file 3: Table S3 and Additional file 8: Table S7). *Draconibacterium orientale* belonged to the family *Draconibacteriaceae* (it was transferred to family *Prolixibacteraceae* in 2014) within the order *Marinilabiliales* [19] and the phylum Bacteroidetes, which are commonly assumed to be specialized in degrading high-molecular-weight compounds in marine environments [26]. *Marinilabiliales* contains four families (*Marinilabiliaceae*, *Prolixibacteraceae*, *Marinifilaceae*, and *Salinivirgaceae* [27]) and has approximately 45 species (Additional file 8: Table S7). Approximately 70% of species were isolated after the so-called enrichment treatment (Additional file 8: Table S7).

In our previous study, we identified 12 novel species in the order *Marinilabiliales* [19], and 10 of those 12 species were isolated using enrichment methods (Additional file 8: Table S7). These results showed that enrichment culturing might be an efficient method for isolating some low-abundance uncultured bacteria by enrichment. Meanwhile, some species, such as *Halomonas denitrificans* and *Marinobacter xestospongiae* (Additional file 5: Table S5), were relatively abundant in the original sediment samples but still recalcitrant to be isolated by direct spread onto MA medium, though enrichment culture could aid their isolation. The results were also consistent with that in *Salinivirga cyanobacteriivorans* isolation [27].

However, during the enrichment culture, some bacteria could be enriched but could not be isolated on the MA medium. One possibility might be due to the different environmental conditions between enrichment and isolation, such as differences in oxygen concentration, liquid enrichment but solid culture, and nutrient composition changes after enrichment. An alternative possibility might have to do with the “Black Queen Hypothesis” proposed by Morris, Lenski, and Zinser [28]. According to this hypothesis, some bacteria, enriched in the enrichment culture, might need to grow close to other bacteria on a petri plate.

We were interested in why some certain groups of bacteria could be enriched and cultured by this method and why such low-abundance strains could be isolated. When most bacteria in energy-limited environments are slow-growing bacteria (“k-strategists”), they may be prepared for a slow but steady existence under the nutrient limitations. Rapid growth is inhibited because the bacteria would not be ready for the end of a brief nutrient flush [29]. Other bacteria, which are rarer in nutrient-limited environments, respond rapidly to nutrient flushes and have a rapid growth when nutrients are plentiful. However, these bacteria (“r-strategists,” such as most *Marinilabiliales* species) must re-enter a dormant state before such a nutrient flush is exhausted. Once such bacteria are in a dormant state, they wait for the next nutrient flush [29] or reaching a VBNC state [12].

As a result, we hypothesized that the enrichment culture in this study helped some species (“k-strategists”) adapt and grow on MA medium and could aid the resuscitation of dormant or VBNC bacteria (“r-strategists”) (Additional file 7: Figure S2). Those resuscitated bacteria were unable to divide during the enrichment culturing but might have been then able to grow on the MA medium. To assess the role of resuscitation, we calculated the proportions of dormant and active taxa in each sample using the (16S rRNA+ 1):(16S rRNA gene + 1) gene ratio and defined any taxon with a ratio > 1 as resuscitation or active [30], owing to the observed low abundance in some bacterial diversity [31]. Active taxa remained 27% of the community in original sediments (Additional file 7: Figure S3), but during enrichment culture, the proportion of active taxa decreased over time (Additional file 7: Figure S3) to 13% of the community. However, order *Marinilabiliales* had considerably higher ratios in enrichment culture compared with original sediments (Fig. 3b). At the family and genus level, *Marinilabiaceae*, *Tangfeifania*, *Draconibacterium*, and *Saccharicrinis* also had considerably higher ratios in enrichment culture (Fig. 3b), suggesting that most bacteria of *Marinilabiliales* were dormant in original sediments and that they could be significantly active after enrichment culturing. In addition, these results were consistent with the idea that certain bacteria were more frequently isolated after enrichment culturing.

We also treated 10 strains that had been isolated but with low abundance in the corresponding enrichment culture stage for induction into a VBNC state (Additional file 7: Figure S4a). Five strains could not grow on MA medium after VBNC induction treatment, even after 10 days of culturing; however, these VBNC cells were resuscitated and formed a colony on MA medium after an enrichment culture treatment (Additional file 7: Figure S4b). The other five strains, which were not fully in the VBNC state under our experimental conditions, were also cultured after enrichment, and their numbers of CFUs were significantly increased by enrichment (Additional file 7: Figure S4b). Real-time PCRs showed that most of the resuscitated strains were not enriched during the enrichment culturing (Additional file 7: Figure S4c). These results suggested that the enrichment culture method included important mechanisms for “resuscitating” some marine sediment bacteria.

### Metatranscriptomic and comparative genomic analyses of the resuscitation mechanism during enrichment culturing

#### Overview

To determine the in situ metabolic activities of the cultured microbes and to explore the resuscitation mechanism in the enrichment mixed culture system, we used metatranscriptomic and comparative genomic analyses of the bacterial metabolic activity in the enrichment system.

**Fig. 4:**
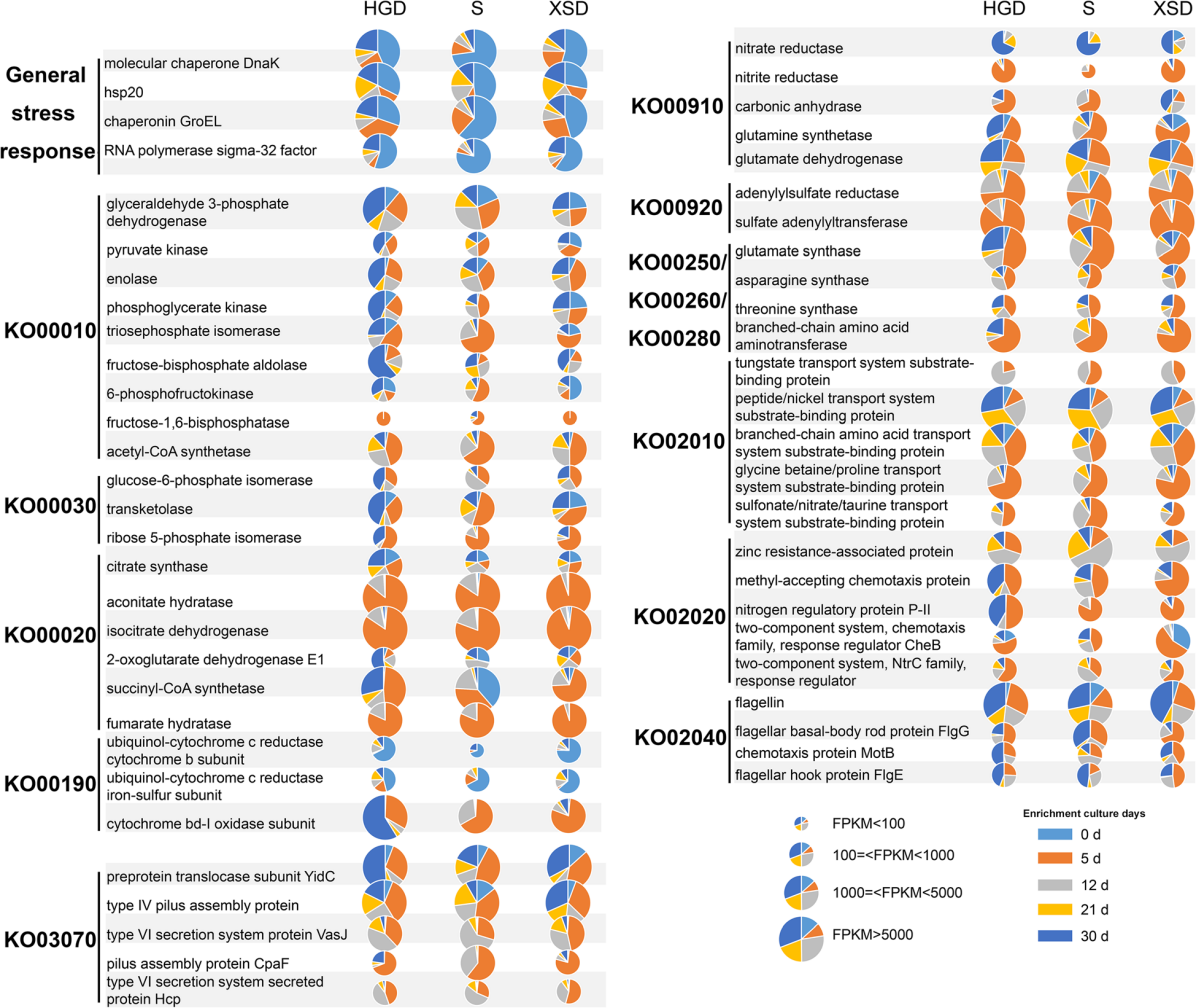
Relative abundances of transcripts associated with the in situ functional activities of the three sediment sample communities receiving enrichment treatment. Genes involved with environment stress resistance, glycolysis/gluconeogenesis (KO00010), the citrate cycle (TCA cycle, KO00020), the pentose phosphate pathway (KO00030), oxidative phosphorylation (KO00190), nitrogen metabolism (KO00910), sulfur metabolism (KO00920), amino acid metabolism (KO00250, KO00260, KO00280), ABC transporters (KO02010), two-component systems (KO02020), flagellar assembly (KO02040), and the bacterial secretion system (KO03070). Light blue indicates the control treatment, and orange indicates 5 days of enrichment culturing. Gray indicates 12 days of enrichment culturing, yellow indicates 21 days of enrichment culturing, and dark blue indicates 30 days of enrichment culturing. The size of a pie slice indicates the total fragments per kilobase of transcript per million fragments mapped (FPKM) of the corresponding gene in each sediment sample. HGD, S, and XSD indicate the three marine sediment samples

For each of the 15 enrichment samples, 2.4 to 6.8 Gb of cDNA were sequenced and passed through quality filters, and the general features of these sequences are shown in Additional file 9: Table S8. Genes involved in ribosomal structure and biogenesis, energy production, translation, amino acid carbohydrate metabolism, and chaperones dominated the transcript pools of all analyzed communities (Fig. 4, Additional file 7: Figure S5). The dominance of the transcripts was similar with results from previous metaproteomic analyses [32–34], indicating the uniformly high abundance of transcripts for the maintenance of basic cellular machinery, growth, and metabolism in normal and extreme environments [35]. As expected, communities from three sediment samples receiving certain enrichment treatments exhibited similar metabolic potentials, reflecting their relatively similar environmental conditions and community compositions (Additional file 7: Figure S1e).

#### Stress responses

**Fig. 5:**
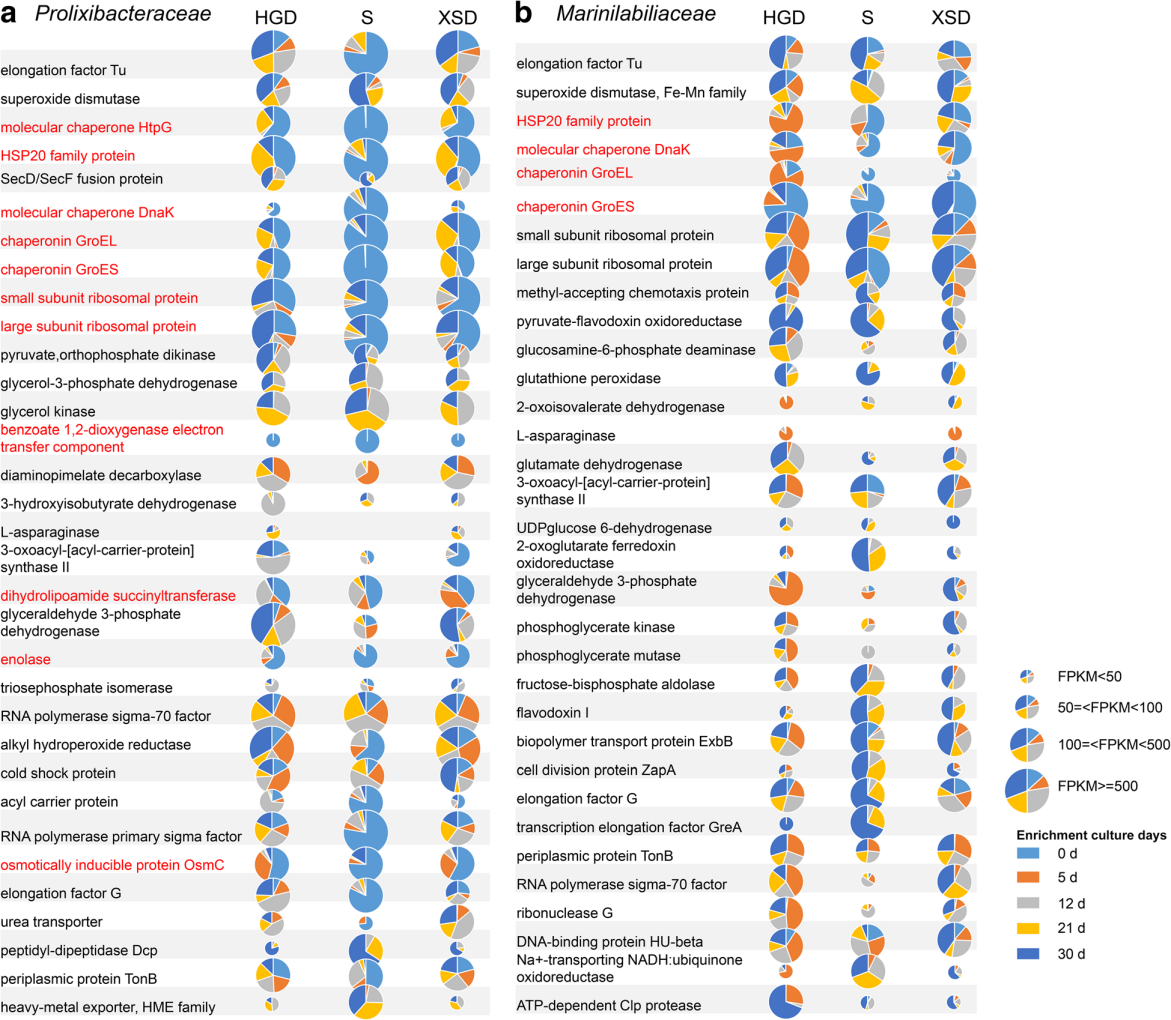
Comparative transcriptional activities of *Prolixibacteraceae* and *Marinilabiliaceae* microbes in the three sediment sample communities under enrichment treatment. The red font indicates that the genes have a significantly high expression in the control treatment. Light blue indicates the control treatment, while the orange indicates 5 days of enrichment culturing. Gray indicates 12 days of enrichment culture, yellow indicates 21 days of enrichment culturing, and dark blue indicates 30 days of enrichment culturing. The size of a pie slice indicates the total fragments per kilobase of transcript per million fragments mapped (FPKM) of the corresponding gene in each sediment sample. HGD, S, and XSD indicate the three marine sediment samples. *Marinifilaceae* respond similarly to the enrichment culture as *Prolixibacteraceae* and *Marinilabiliaceae*, and detailed information is shown in Additional file 10: Table S9

Some genes that participate in general stress responses were upregulated in the control samples, including genes encoding the molecular chaperone DnaK, small heat shock proteins (sHsp20), the molecular chaperone IbpA, the ATP-dependent Clp protease ClpB, the chaperonin GroEL, and the chaperonin GroES (Fig. 4). In *E. coli*, GroEL assists the folding of nascent or stress-denatured proteins [36], and small heat shock proteins (sHsp20 and IbpA) are molecular chaperones that prevent the aggregation of non-native proteins [37]. Genes encoding transcriptional regulation (RNA polymerase sigma-32 factor) and sulfur metabolism (sulfite reductase, adenylylsulfate reductase) were also upregulated in control samples (Fig. 4). Sigma-32 factor is important for transcriptional regulation under stress [38]. Metatranscriptomic analysis of *Marinilabiliales* microbes was consistent with this result and showed that before enrichment culturing, genes that participate in general stress responses, such as those encoding sHsps20, DnaK, GroEL, enolase, and OsmC (Fig. 5; Additional file 10: Table S9), were also upregulated. These genes were reported to be significantly upregulated in various VBNC bacteria and had essential roles in VBNC bacteria survival [39–41]. Most importantly, considering that most *Marinilabiliales* microbes were not directly isolated on nutrient agar medium from the original marine sediment (Additional file 8: Table S7), and these microbes probably maintain dormancy in marine sediment (Fig. 3b), these results suggested that most bacteria in original marine sediments are under stress or might even be in the dormant state [31], and these issues make the direct culturing of microbial dark matter difficult.

#### Cellular metabolism

During enrichment culturing, most of these general stress response genes were downregulated (Figs. 4 and 5). In contrast, genes that participate in aspects of cellular metabolism such as the citrate cycle (TCA cycle), glycolysis/gluconeogenesis, the pentose phosphate pathway, nitrogen metabolism, oxidative phosphorylation, sulfur metabolism, and amino acid metabolism were upregulated, suggesting the microbes have high metabolic activity (Fig. 4). Carbohydrate metabolism was one of the most abundantly represented categories and has a key role in cell growth [42]. Expression of genes involved in most of the important steps in glycolysis was detected, and the expression of several glycolysis genes increased significantly in all three sediment samples during enrichment culturing (Fig. 4). The gene encoding acetyl-CoA synthetase was significantly upregulated during the 5 days of enrichment culturing, probably because we added 0.2% CH_3_COONa, which becomes a substrate of acetyl-CoA synthetase, in the broth before enrichment. Another way to enhance acetyl-CoA production was using the oxidative decarboxylation of pyruvate, which is generated from glycolysis, and the expression of genes related to the pyruvate dehydrogenase system was also significantly upregulated in the first stage (5 days) of enrichment. Acetyl-CoA is a molecule that participates in many biochemical reactions in protein, carbohydrate, and lipid metabolisms [42]. Its main function is to deliver the acetyl group to the TCA cycle to be oxidized for energy production. CH_3_COONa and acetyl-CoA might thus have potential roles in resuscitation; however, the detailed mechanisms of this process should be examined in our further studies.

In all three sediment samples, the expression of genes involved in the first half of the TCA cycle during different enrichment stages (range of 1.5-fold and 1000-fold) greatly increased, leading to synthesis of 2-oxoglutarate, a key metabolite in the regulation of carbon and nitrogen metabolism [43] (Fig. 4). The genes related to carbohydrate metabolism were also upregulated. The aerobic respiration pathway genes (the ubiquinol-cytochrome c reductase iron-sulfur subunit and the ubiquinol-cytochrome c reductase cytochrome b subunit) were highly expressed in the day 0 treatment samples, while anaerobic respiration pathways (the cytochrome bd-I oxidase subunit) were upregulated with prolonged enrichment culturing (Fig. 4), suggesting that the conversion of aerobic respiration to anaerobic respiration occurred in this enrichment culture. Genes related to the biosynthesis and degradation of L-serine (D-3-phosphoglycerate dehydrogenase, SerA, more than 100-fold after 5 days of enrichment culturing) and L-threonine (threonine synthase, ThrC, more than 100-fold) were upregulated in the enrichment culture (Fig. 4). Catabolism of l-serine and l-threonine was reported to provide *E. coli* cells with a source of energy under anaerobic conditions [44]; enhancement of production and degradation of l-serine and l-threonine is thus probably also a mode of energy generation used by microbes under the anaerobic conditions caused by this anaerobic enrichment culture method.

Likewise, glutamine synthetase gene (*glnA*) and glutamate synthase gene (*gltBD*), which encoding the enzymes permitted the incorporation of ammonium into glutamine and utilization of glutamate [45], were regulated (Fig. 4). Two-component system-related nitrogen assimilation (NtrC family) and peptide and amino acid transport systems (ABC transporters) were also highly upregulated (Fig. 4), suggesting that populating microorganisms had massive demand for nitrogen resources. However, genes encoding enzymes for the utilization of nitrate and nitrite (for example, nitrate reductase and nitrite reductase) exhibited relatively low transcriptional activities, and nitrate reductase was highly expressed during only the late state of enrichment culturing (Fig. 5). These results showed that the enrichment culture communities might acquire nitrogen resources via the direct transformation and utilization of organic nitrogen sources. In addition, genes involved in sulfate reduction, such as those encoding adenylyl-sulfate reductase [46], were also enriched, which suggests that the main sulfur metabolism in the microbiota might change during resuscitation.

Bacteria within the *Marinilabiliales* group also showed highly active cellular metabolism in the enrichment culture. Genes encoding pyruvate phosphate dikinase, glycerol-3-phosphate dehydrogenase, glyceraldehyde 3-phosphate dehydrogenase, glycerol kinase, pyruvate-flavodoxin oxidoreductase, and a cell division protein (ZapA) were upregulated (Fig. 5). Pyruvate phosphate dikinase is a key enzyme in gluconeogenesis and enhances the survival of bacteria [47]. Glycerol-3-phosphate dehydrogenase serves as a major link between carbohydrate metabolism and lipid metabolism. Glyceraldehyde 3-phosphate dehydrogenase and glycerol kinase are enzymes that participate in glycolysis. Pyruvate-flavodoxin oxidoreductase participates in the oxidative decarboxylation of pyruvate to acetyl-CoA. Most of those genes affect pyruvate metabolism are related to carbohydrate and energy metabolism and aid resuscitation from the VBNC state [40], suggesting *Marinilabiliales* microbes were resuscitated from non-culturable (dormant) states, and the results were consistent with the 16S rRNA:16S rRNA gene analysis (Fig. 3b). We added 0.11% sodium pyruvate to the enrichment culture medium in this study, and the results was consistent with a study that showed that pyruvate might be a key molecule in resuscitation by converting bacteria from a non-culturable state to a growing and colony-forming state [22].

#### Competition strategy

Genes involved in flagellar assembly and type IV and type VI secretion systems (T4SSs and T6SSs) were upregulated, especially during the late stages of enrichment culturing (21 and 30 days of treatment) (Fig. 4). With the dynamic changes in bacterial communities in this enrichment mix culture, the T6SSs are among the mechanisms that aid in the struggle against other bacterial species [48]. T6SSs participate in a broad variety of functions, including virulence, antibacterial activity, and conquering new territory [49]. Similarly, T4SSs represent a highly diverse superfamily of secretion systems found in many bacterial species and also can deliver a killing toxin to bacterial neighbors so the organism persists in an ecological niche [50], suggesting that an increase in cell density increases the concentration of T4SS and T6SS components in bacteria and the capacity of the cell to win the competition. These results also support the ability of novel species *Bradymonas sediminis* FA350 and *Bradymonas* sp*.* B210 (with T4SSs) to prey on other bacteria and to reach a relatively high growth rate after enrichment culturing (data not shown).

#### Cooperation or coordination strategy

**Fig. 6:**
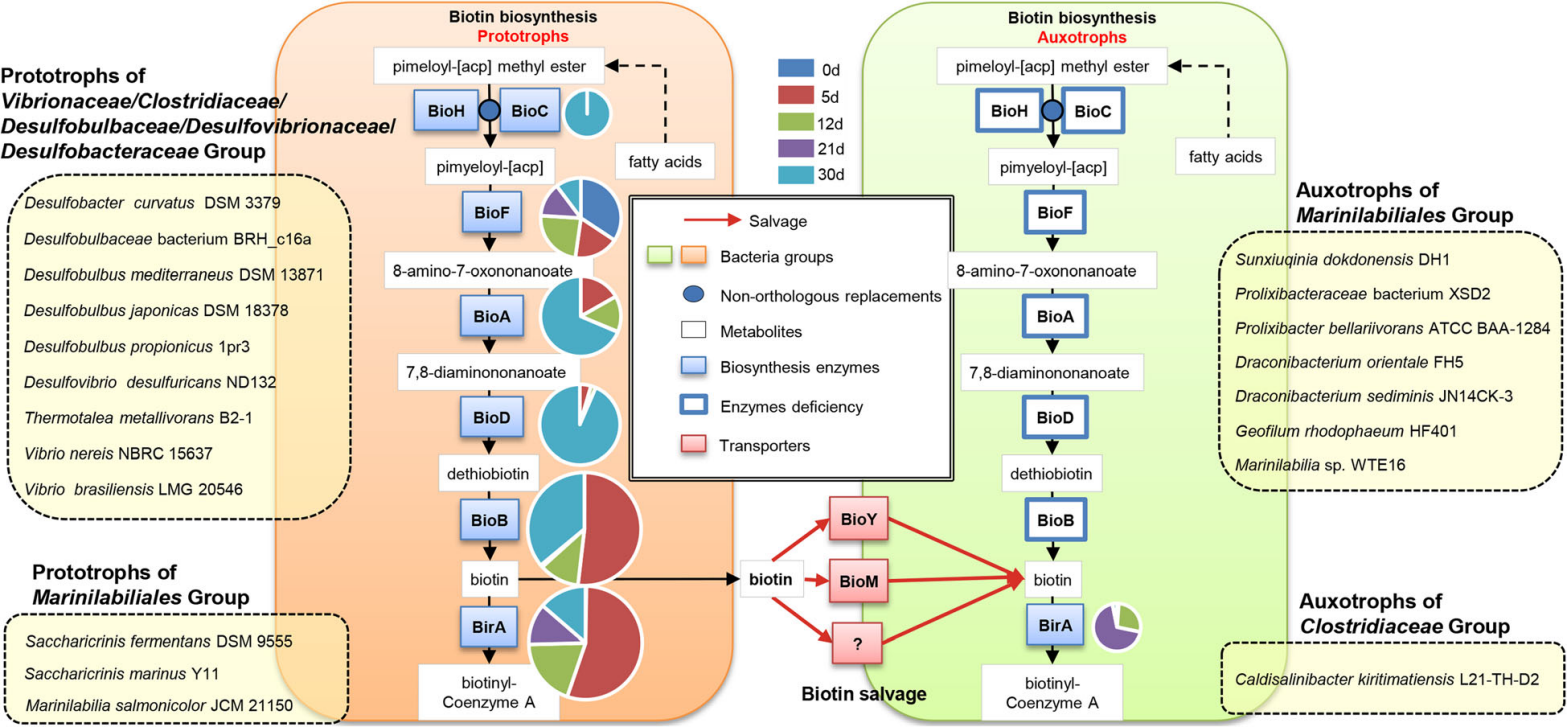
Comparative genomics analysis of correlations between *Marinilabiliales* microbes and the microorganisms enriched during enrichment culturing. Overview of biotin biosynthesis and precursor salvage. Biosynthetic enzymes: BioH, pimeloyl-[acyl-carrier protein] methyl ester esterase; BioC, malonyl-CoA O-methyltransferase; BioF, 8-amino-7-oxononanoate synthase; BioA, adenosylmethionine-8-amino-7-oxononanoate aminotransferase; BioD, dethiobiotin synthetase; BioB, biotin synthase; and BirA, biotin-[acetyl-CoA-carboxylase] ligase. Biotin translocases include the BioYM ECF-type transporter. The auxotrophic groups, which mainly contain *Marinilabiliales* bacteria, are unable to synthesize biotin de novo and are missing most of the related pathway genes. Phototrophic groups, which mainly contain *Vibrionaceae*, *Clostridiaceae*, *Desulfobulbaceae*, *Desulfovibrionaceae*, and *Desulfobacteraceae* bacteria, can synthesize biotin de novo. The *Desulfobacteraceae* node has no correlations with the *Prolixibacteraceae* node and the *Marinilabiliaceae* node; however, the bacteria within this family were highly enriched and became the dominant family during enrichment culturing. We also analyzed the genomes of this family’s members. Pie slices indicate the total fragments per kilobase of transcript per million fragments mapped (FPKM) of the corresponding gene in each sediment sample

To comprehensively investigate the interactions between microbial communities during enrichment culturing, we conducted network analysis (Additional file 11: Table S10), and the results showed that during the enrichment culturing, *Marinilabiliaceae* levels were positively correlated with *Vibrionaceae*, *Desulfovibrionaceae*, *Kazan*-2B-17, and *Clostridiaceae* content and negatively connected with the *Lentimicrobiaceae* and *Flavobacteriaceae* contents (Additional file 11: Table S10). *Prolixibacteraceae* had positive connections with *Desulfobulbaceae* and was negatively connected with *SAR406_clade* (Additional file 11: Table S10). Most microorganisms in environments are auxotrophs, thus relying on external nutrients for growth, such as the exchange of vitamins [51]. Several abundant bacteria in the gut (for example, Bacteroides spp.) are unable to synthesize essential vitamins such as cobalamin (vitamin B_12_) [52]. Some members of the Bacteroidetes phylum are lacking of some of the genes that are necessary for the synthesis of VB_12_. As a result, to explore whether the connections between groups related to the nutrient exchange then affected the culturability of bacteria such as *Marinilabiliales* (belonging to the Bacteroidetes phylum) during enrichment culturing, we used a subsystem approach and comparative genomic analysis to reconstruct amino acid, vitamin, and cofactor biosynthetic pathways and transport capabilities in 20 genomes, which included 10 genomes from the *Marinilabiliales* group (5 genomes acquired from this study and 5 from public database) and 10 genomes (retrieved from public database) of enriched organisms having positive connections with the *Marinilabiliales* group during enrichment culturing (Additional file 12: Table S11). The comparative genomic analysis showed that both groups could synthesize most essential amino acids (such as glutamine, glutamate, lysine, and threonine biosynthesis) and B vitamin-related enzyme cofactors (folate, thiamine, flavodoxin, and pyridoxin) de novo. However, most microorganisms within the *Marinilabiliales* group are unable to synthesize biotin and cobalamin de novo (Fig. 6, Additional file 7: Figure S6, Additional file 12: Table S11), while members of the other group had this ability. Genes involved in biotin and cobalamin biosynthesis of prototrophic bacteria, which had positive connections with *Marinilabiliales*, were upregulated during enrichment culturing (Fig. 6 and Additional file 7: Figure S6), suggesting that these enriched microorganisms might generate biotin and cobalamin during enrichment culturing and assist in the resuscitation of *Marinilabiliales* microbes from a non-culturable state or at least contribute to the shared pool of cofactors when they lyse. The availability of cofactors or precursors in the pool can also be controlled by the metabolic status and export capabilities of the respective prototrophs (Fig. 6 and Additional file 7: Figure S6). Furthermore, because certain processes that depend on cofactors are essential, they can serve as control points for the coordination of community member abundance and function, suggesting that the division of labor in cofactor production is closely tied to emergent community properties. These results were similar to those obtained from the human gut microbiome [53], free-living bacteria in aquatic systems [54], and unicyanobacterial consortia (UCC) [55], microbial communities in which syntrophic metabolism of essential enzyme cofactors was found. These interactions can promote positive, negative, or neutral effects on the fitness of the community members, resulting in complex relationships. Temporal shifts in the community composition strongly affect the interactions between cooperative bacteria [51]. Diversification and specialization of precursor salvage provides a mechanism for the division of labor in microbial communities and a selective advantage for partners uniquely suited for optimal syntrophic biosynthesis of cofactors [56]. However, the comparative genomic analysis based on the published genomes was a kind of possible speculation, and some of related genomes with different original habitat might have some differences in particular pathways. Over all, these findings have significant implications for cultivation of environmental microbes and when cultivating uncharacterized microbes, we suggest adding standard vitamin supplements.

### Target bacterial culture based on the enrichment culture method

Because most of the bacteria whose abundance increased during enrichment culturing were strictly anaerobic bacteria (Additional file 7: Figure S1e), we also designed an anaerobic culture strategy using the broad-range medium MA with NO_3_^−^ and vitamin B complex to specifically select for anaerobic or facultative anaerobic bacteria. The almost full-length 16S rRNA gene library sequencing was performed for all of the colonies on the plates under anaerobic culture conditions to analyze the microorganism groups in the anaerobic culture system. Many novel anaerobic or facultative anaerobic species were obtained by this method (Additional file 13: Table S12). Some novel species within the order *Marinilabiliales* could be isolated at high frequencies due to their abundant OTUs on the MA medium (Additional file 13: Table S12). However, the enrichment culture method is currently not perfect due to the unique culture conditions required by some taxa. To understand and manipulate the microbiome, researchers must dissect and engineer the interactions within these critical communities [57].

## Conclusions

Our workflow enables large-scale culturing, archiving, genomic sequencing, and phenotyping of novel bacteria from marine sediment microbiotas, which were previously considered recalcitrant. Degree of enrichment analysis, dormant and active taxa calculation, VBNC bacteria resuscitation analysis, combined with metatranscriptomic and comparative genomic analyses of the culturability mechanism during enrichment culture showed that the so-called enrichment method could isolate “recalcitrant” strains not only through enriching their abundance but also through resuscitation. In addition, the enrichment culture is a complicated mixed culture system that utilizes a competition, cooperation, or coordination strategy among bacterial communities. Considering that cultivation techniques must evolve from axenic to mixed cultures, comprehending microbial world [16], we should redevelop an understanding of the classic enrichment culture method. In addition, enrichment culture methods can be developed and used to construct a model for analyzing mixed cultures and exploring microbial dark matter. This simple method still has great potential for cultivating recalcitrant microorganisms. Our streamlined, single-medium approach builds on the considerable efforts of others [4, 9, 11, 58] and provides a new approach to mining marine microbial dark matter based on resuscitation in mixed cultures. In future studies, we hope to develop a series of enrichment culture methods to form a multi-type “enrichment culturomics,” which will then be combined with the current “culturomics” [59] to reveal many more uncultured microbes.

## Methods

### Sediment collection and bacterial isolation

Three sediment samples were collected from the intertidal zone of Weihai, China (HGD 37°32′0″ N 122°03′59″ E, XSD 37°31′17″ N 122°01′8″ E, and S 37°28′45″ N 121°57′15″ E) on 10 May 2016. Sediment samples were collected by a surface sediment sampler. Following collection, samples were placed in sterile 50 ml plastic Whirl-Pak bags (NASCO, Modesto, CA, USA) and kept cool until they were processed (within 4 h) using the following procedures.

The medium for enrichment culture was a low-nutrient medium that consisted of the following ingredients in seawater: 0.1% NH_4_Cl, 0.2% CH_3_COONa, 0.02% MgSO_4_·7H_2_O, 0.02% yeast extract, 0.02% peptone, 0.1% EDTA-Na_2_, and 0.11% sodium pyruvate. The pH of the medium was adjusted to 7.5 and then autoclaved. A 10% (*w*/*v*) NaHCO_3_ solution was filtered, and a 2% (*w*/*v*) KH_2_PO_4_ solution was autoclaved. Each solution was added to the autoclaved media (10 ml per liter).

Enrichment culture incubation was performed at 25 °C for 0, 5, 12, 21, and 30 days in separate 500 ml sealed glass bottles (filled with medium and 20 g of sediment sample). A detailed protocol is described in the Extended Experimental Procedures.

### Culture-independent microbial community composition

We extracted DNA and RNA from enriched samples (from 0 to 30 days of incubation) using commercially available kits and protocols (PowerSoil DNA Isolation Kit and further purification using a Power Clean ProDNA Clean-up Kit from MoBio for DNA, and an RNeasy isolation kit from Qiagen for RNA). Total RNA quality was checked with a Bioanalyzer 2100 (Agilent Technologies, Santa Clara, CA, USA). DNA contamination was analyzed by PCR with universal archaeal and bacterial 16S rRNA gene primers and ethidium bromide agarose gel electrophoresis. When DNA was present, it was removed by DNase I treatment (TaKaRa, China) for 30 min at 37 °C, followed by lithium chloride/EtOH precipitation overnight at − 20 °C [60]. We synthesized cDNA from RNA using random hexamer primers and a SuperScript III first-strand synthesis kit (Invitrogen). PCR amplification, purification, and pooling and the subsequent pyrosequencing of a region of the 16S rRNA gene were performed following the procedure described by Fierer et al. [61]. We used the primer set composed of 515F (5′-GTGCCAGCMGCCGCGG-3′) and 907R (5′-CCGTCAATTCMTTTRAGTTT-3′), which was designed to amplify the V4–V5 region and was demonstrated in silico to be universal for nearly all bacterial taxa. Sequencing was carried out on a MiSeq platform at the Majorbio Bio-Pharm Technology Co., Ltd. (Shanghai, China). Detailed protocols are described in the Extended Experimental Procedures.

To assess the proportion of dormant and active taxa during enrichment culturing, we calculated the 16S rRNA+ 1:16S rRNA gene + 1 ratio for each taxa in each sample and defined a taxon as active with a ratio > 1 [30, 31]. Implicit in this calculation is the assumption that if a 16S rRNA sequence is present in the RNA portion of a sample, it must also be present at least one time on the DNA portion but was not detected due to incomplete sequencing. To assess the diversity of active taxa, we calculated the 16S rRNA:16S rRNA gene ratio for each taxon and defined a taxon as active when its ratio was > 1.

### Degree of enrichment detection

The dataset was normalized by random resampling, based on smallest sample size (*n* = 50,443 sequences in this study). To detect the abundance of the isolated strains in each enrichment period, local BLAST analysis of the 16S rRNA gene of isolated strains and 16S rRNA gene libraries of different stages of enrichment from MiSeq results were conducted using NCBI local BLAST, and 16S rRNA gene libraries with 99% sequence similarity (*E* value<1e-50) were considered the same species.

To assess the degree of enrichment, we calculated the “enriched 16S rRNA gene (abundance of corresponding isolated sample)+1”:“16S rRNA gene (abundance of 0 day)+1” ratio for each taxon in each sample. When the ratio > 1, the species was enriched when it was isolated. In contrast, a ratio ≤ 1 indicates that the species was not enriched when it was isolated. The “+1” is a correction calculation to prevent the error caused by an initial abundance of 0 because an isolated species could have at least 1 read in the initial sample and the isolated sample.

### rRNA removal, cDNA library construction, and transgenomics sequencing

The total RNA of samples was subjected to an rRNA removal procedure using a Ribo-zero Magnetic kit according to the manufacturer’s instructions (Epicentre, an Illumina® company). Next, cDNA libraries were constructed using a TruSeq™ RNA sample prep kit (Illumina). Barcoded libraries were paired-end sequenced on an Illumina HiSeq 2500 platform at the Majorbio Bio-Pharm Technology Co., Ltd. (Shanghai, China) using a HiSeq 4000 PE Cluster Kit and a HiSeq 4000 SBS Kit according to the manufacturer’s instructions (www.illumina.com). Detailed protocols are described in the Extended Experimental Procedures.

### Induction of VBNC state and resuscitation of growth

The selected cells were cultured in marine agar 2216 (MA; BD) broth at 30 °C for 24 h with mild shaking at 150 rpm and were harvested by centrifugation at 16,000×*g* for 3 min. The bacterial cells were washed twice with sterile seawater, harvested again by centrifugation at 16,000×*g* for 3 min and finally resuspended in sterile seawater to a density of 10^8^ CFU/ml. The cells were then stored at 4 °C for 35 days. The bacterial cell state was monitored over time using the dilution plate count method on MA medium. The VBNC cells were the bacteria that could not form colonies on MA after such treatment. The experiments were conducted in triplicate.

The resuscitation of VBNC cells used the enrichment culture method. VBNC cells were incubated in enrichment culture medium at 25 °C for 5 days, spread onto MA and incubated at 28 °C for 1 day. Culturable cells were enumerated based on the formation of visible colonies on MA medium. VBNC cells directly spread onto MA, incubated at 28 °C for 6 days, were used as a control treatment.

### Quantitative real-time PCR

The enumeration of each of the selected bacterial 16S rRNA gene copies of VBNC samples before and after enrichment culture of 5 days was performed via quantitative real-time PCR to evaluate the cell number during the enrichment culture. Gene copy numbers of VBNC sample enrichment cultures at 0 days were used as controls. We used the primer pair composed of 341F (5΄-CCTACGGGAGGCAGCAG-3΄) and 534R (5΄-ATTACCGCGGCTGCTGGCA-3΄) for detection and quantification [62]. Quantification was based on the fluorescence intensity of SYBR green dye, and reactions for each sample were carried out in an ABI StepOnePlus thermal cycler. The reactions were performed in a total volume of 20 μl using Absolute QPCR SYBR Green Rox Abgene/Thermo (Bio-Medicine), 1 μM of each primer, and 10 ng of sample DNA, which was added to each reaction to reach a final concentration of 0.025 μg/μl. The bacterial 16S rRNA gene fragments were amplified using an initial denaturation step at 95 °C for 15 min and then 35 cycles of 15 s at 95 °C, 30 s at 60 °C, 30 s at 72 °C, and 30 s at 80 °C [63]. Then, the Plasmid DNA Standard was constructed by introducing the 16S rDNA gene amplified from *Bacillus subtilis* into the pMD19 T-Vector (TaKaRa) according to the manufacturer’s instructions. The Plasmid DNA Standard was then gel-purified and quantified. DNA copy number was determined by the concentration and relative molecular weight of the Plasmid DNA. Then, dilutions were made with EASY dilution solution (TaKaRa) to produce concentrations ranging from 1 × 10^7^ to 1 × 10^1^ DNA copies/μl to generate calibration curves. In this study, results with Cq values greater than 35 were considered negative. Error bars in diagrams represent standard deviations from three independent experiments.

### Generation of interaction networks

The network analyses were performed using the Molecular Ecological Network Approach (MENA) [64] pipeline, which was used to generate interaction networks. Read counts were filtered manually to exclude OTUs with > 2 “zero” occurrences across 15 samples and were uploaded to the pipeline. The majority was set to one (a parameter means that only OTUs > 1 could be kept for further analysis), missing data were kept blank, read counts were converted by logarithm, and the Pearson correlation coefficient was used as the similarity measure. To make the correlations more stringent and clearer, we set the threshold value ≥ |0.7| [65].

### Comparative genomic analysis of vitamin-related cofactor biosynthesis pathways

To reveal the potential relationships between the *Marinilabiliales* species and the interworking groups, we used the taxonomy annotation of metatranscriptome sequences to search for target species, and selected the abundant taxonomy annotation as the reference species. It is hard to define all the exact interworking strains and the genomes during the enrichment culture. Therefore, we used some of the published genomes as the representatives. Along with 5 genomes obtained from this study, totally 20 genomes, which included 10 genomes of organisms within the *Marinilabiliales* group and 10 genomes of enriched organisms having positive connections with the *Marinilabiliales* group, were used to make the comparative genomics analysis. (Additional file 12: Table S11). Genome-based analysis of B vitamin-related cofactor biosynthesis pathways in 20 genomes of bacteria was performed using the subsystem-based comparative genomic approach [55] implemented in SEED/RAST [66, 67] combined with the genomic reconstruction of vitamin-specific transcriptional regulons and the identification of candidate vitamin transporters, as previously described [68]. All of the genomes can be accessed via NCBI (https://www.ncbi.nlm.nih.gov/genome/), and the detailed accession numbers are shown in Additional file 12: Table S11.

Extensive manual pathway curation was assisted by KEGG orthology assignments from the BlastKOALA annotation tool [69]. Cofactor requirements were asserted by cataloging respective enzymes from annotated genomes and connecting them to metabolic pathways in KEGG [69] and SEED [66].

### Data availability

The 16S rRNA gene and 16S rRNA data sets have been deposited in the Sequence Read Archive under accession numbers SRP132130 and SRP133458 for all the samples. The accession numbers of full-length 16S rRNA gene data sets for the five anaerobic cultured plates were SRP132148. The metatranscriptomic sequences for all the samples have been deposited in the Sequence Read Archive under accession numbers SRP133988. All bacterial isolates have been deposited at the Shandong Infrastructure of Marine Microbial Resources hosted by the Laboratory of Marine Microbiology at Shandong University (http://www.sdum.wh.sdu.edu.cn/search.html?itemId=14). The accession numbers of the isolated strains are listed in Additional file 3: Table S3. Any isolates with accession numbers are available upon request.

## Additional files

Additional file 1: **Table S1.** Cultivable bacterial numbers at different periods of enrichment-culture. (DOCX 127 kb)

Additional file 2: **Table S2.** Number of different phylum/class strains cultured from S sample. (DOCX 18 kb)

Additional file 3: **Table S3.** The detailed information of each species on phylogenetic tree. (XLSX 63 kb)

Additional file 4: **Table S4.** Species on phylogenetic tree isolated from different sediment samples. (XLSX 15 kb)

Additional file 5: **Table S5.** The recruited reads of all isolates in three sediment samples of enrichment culture. (XLSX 26 kb)

Additional file 6: **Table S6.** Mean bacteria diversity of the samples. (XLSX 10 kb)

Additional file 7: The Additional methods and additional figures. (DOCX 1903 kb)

Additional file 8: **Table S7.** The detailed information for all validly published species in *Marinilabiliales*. (XLSX 13 kb)

Additional file 9: **Table S8.** The general features of metatranscriptomic sequencing of the 15 samples. (XLSX 9 kb)

Additional file 10: **Table S9.** Detailed information of genes expression in Marinilabiliales in the 15 samples. (XLSX 387 kb)

Additional file 11. **Table S10.** Detailed information of the network analysis. (XLS 42 kb)

Additional file 12: **Table S11.** The general features of the 20 selected genomes and the biotin/VB12 biosynthesis pathway analysis. (XLSX 17 kb)

Additional file 13: **Table S12.** The isolates diversity on MA under anaerobic culturing. (XLS 84 kb)
