# Supplementary material for: Metatranscriptomic and comparative genomic insights into resuscitation mechanisms during enrichment culturing
Source: Microbiome. 2018 Dec 26;6:230. doi: 10.1186/s40168-018-0613-2 (PMC6307301; doi:10.1186/s40168-018-0613-2)
Supplement: Supplementary file 1 — Table S1. Cultivable bacterial numbers at different periods of enrichment-culture. (DOCX 127 kb) [file 40168_2018_613_MOESM1_ESM.docx]

Table S1 Cultivable bacterial numbers at different periods of enrichment-culture

| Sample | Enrichment-culture (days) | Cultivable bacterial number (/ml) | Isolates number |
| --- | --- | --- | --- |
| XSD0 | 0 | 2×10^6^ | 121 |
| XSD1 | 5 | 1×10^7^ | 110 |
| XSD2 | 12 | 7×10^6^ | 80 |
| XSD3 | 21 | 7×10^6^ | 65 |
| XSD4 | 30 | 4×10^6^ | 55 |
| HGD0 | 0 | 1×10^6^ | 107 |
| HGD1 | 5 | 9×10^7^ | 88 |
| HGD2 | 12 | 4×10^7^ | 45 |
| HGD3 | 21 | 4×10^7^ | 41 |
| HGD4 | 30 | 6×10^6^ | 58 |
| S0 | 0 | 3×10^6^ | 123 |
| S1 | 5 | 3×10^8^ | 121 |
| S2 | 12 | 7×10^7^ | 50 |
| S3 | 21 | 4×10^7^ | 45 |
| S4 | 30 | 2×10^7^ | 132 |
| 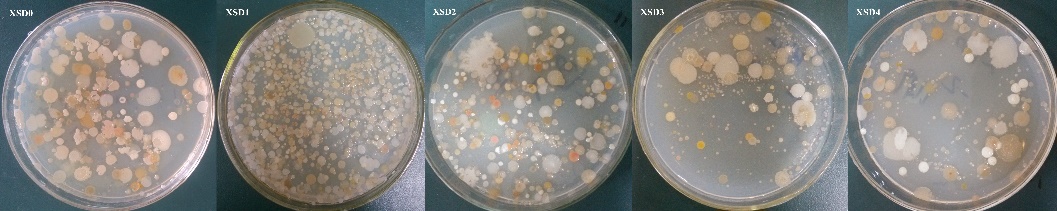 | | | |
