# Supplementary material for: Metatranscriptomic and comparative genomic insights into resuscitation mechanisms during enrichment culturing
Source: Microbiome. 2018 Dec 26;6:230. doi: 10.1186/s40168-018-0613-2 (PMC6307301; doi:10.1186/s40168-018-0613-2)
Supplement: Supplementary file 2 — Table S2. Number of different phylum/class strains cultured from S sample. (DOCX 18 kb) [file 40168_2018_613_MOESM2_ESM.docx]

Table S2-1 Number of different phylum/class strains cultured from S sample

|  | | S0 | | S1 | | S2 | | S3 | | S4 | |
| --- | --- | --- | --- | --- | --- | --- | --- | --- | --- | --- | --- |
| Bacteroidetes | | 15 | | 27 | | 27 | | 33 | | 18 | |
| Firmicutes | | 33 | | 6 | | 12 | | 3 | | 3 | |
| Actinobacteria | | 15 | | 9 | | 9 | | 15 | | 0 | |
| Proteobacteria | α- *proteobacteria* | 69 | 39 | 42 | 12 | 39 | 21 | 30 | 9 | 21 | 15 |
|  | γ- *proteobacteria* |  | 30 |  | 27 |  | 15 |  | 15 |  | 6 |
|  | ε- *proteobacteria* |  | 0 |  | 3 |  | 3 |  | 3 |  | 0 |
|  | δ- *proteobacteria* |  | 0 |  | 0 |  | 0 |  | 3 |  | 0 |
| Summary | | 132 | | 84 | | 87 | | 81 | | 42 | |

Table S2-2 Number of different phylum/class strains cultured from XSD sample

|  | | XSD0 | | XSD1 | | XSD2 | | XSD3 | | XSD4 | |
| --- | --- | --- | --- | --- | --- | --- | --- | --- | --- | --- | --- |
| Bacteroidetes | | 12 | | 12 | | 12 | | 11 | | 15 | |
| Firmicutes | | 66 | | 51 | | 36 | | 22 | | 21 | |
| Actinobacteria | | 0 | | 3 | | 6 | | 3 | | 6 | |
| Proteobacteria | α- *proteobacteria* | 48 | 21 | 10 | 2 | 48 | 9 | 30 | 6 | 30 | 6 |
|  | β- *proteobacteria* |  | 0 |  | 0 |  | 0 |  | 0 |  | 0 |
|  | γ- *proteobacteria* |  | 27 |  | 7 |  | 39 |  | 24 |  | 24 |
|  | ε- *proteobacteria* |  | 0 |  | 1 |  | 0 |  | 0 |  | 0 |
| Summary | | 126 | | 32 | | 102 | | 66 | | 72 | |

Table S2-3 Number of different phylum/class strains cultured from HGD sample

|  | | HGD0 | | HGD1 | | HGD2 | | HGD3 | | HGD4 | |
| --- | --- | --- | --- | --- | --- | --- | --- | --- | --- | --- | --- |
| Bacteroidetes | | 24 | | 15 | | 15 | | 14 | | 10 | |
| Firmicutes | | 75 | | 15 | | 27 | | 10 | | 9 | |
| Actinobacteria | | 27 | | 6 | | 3 | | 3 | | 2 | |
| Proteobacteria | α- *proteobacteria* | 57 | 12 | 27 | 0 | 3 | 3 | 6 | 6 | 15 | 6 |
|  | β- *proteobacteria* |  | 15 |  | 0 |  | 0 |  | 0 |  | 9 |
|  | γ- *proteobacteria* |  | 30 |  | 21 |  | 0 |  | 0 |  | 0 |
|  | ε- *proteobacteria* |  | 0 |  | 6 |  | 0 |  | 0 |  | 0 |
| Summary | | 183 | | 63 | | 48 | | 33 | | 36 | |

**NOTE:** S0-S4, XSD0-XSD4, and HGD0-HGD4 indicate 0, 5, 12, 21, and 30 d of enrichment culturing, respectively.
