# Supplementary material for: Metatranscriptomic and comparative genomic insights into resuscitation mechanisms during enrichment culturing
Source: Microbiome. 2018 Dec 26;6:230. doi: 10.1186/s40168-018-0613-2 (PMC6307301; doi:10.1186/s40168-018-0613-2)
Supplement: Supplementary file 7 — The Additional methods and additional figures. (DOCX 1903 kb) [file 40168_2018_613_MOESM7_ESM.docx]

**Supplementary information**

**Metatranscriptomic and comparative genomic insights into resuscitation mechanisms during enrichment-culture**

**Da-Shuai Mu^1,2^, Qi-Yun Liang^2^, Xiao-Man Wang^2^, De-Chen Lu^2^, Ming-Jing Shi^2^, Guan-Jun Chen^1,2^, Zong-Jun Du^1,2^***

^1^ State Key Laboratory of Microbial Technology, Institute of Microbial Technology, Shandong University, Qingdao 266237, China

^2^ College of Marine Science, Shandong University, Weihai 264209, PR China

*for correspondence. E-mail: duzongjun@sdu.edu.cn

Running title: Enrichment culture of marine microbial dark matter

**Table of contents:**

Extended Experimental Procedures

Supplementary Figure 1

Supplementary Figure 2

Supplementary Figure 3

Supplementary Figure 4

Supplementary Figure 5

Supplementary Figure 6

Table S1: Cultured bacterial numbers at different periods of enrichment-culture

Table S2: Number of different phylum/class strains in three samples

Table S3: The detailed information of each species on phylogenetic tree

Table S4: Species on phylogenetic tree isolated from different sediment samples

Table S5: The recruited reads of all isolates in three sediment samples of enrichment culture

Table S6: Mean bacteria diversity of the 15 samples

Table S7: The detailed information of all cultured species in *Marinilabiliales*

Table S8: The general features of metatranscriptomic sequencing of the 15 samples

Table S9: Detailed information of genes expression in *Marinilabiliales* in the 15 samples

Table S10. Detailed information of the network analysis

Table S11: The general features of the 20 selected genomes and the biotin/VB_12_ biosynthesis pathway analysis

Table S12: The isolates diversity on MA under anaerobic culturing

Supplementary references

**Extended Experimental Procedures**

**Enrichment-culture and isolates identification**

Enrichment culture incubation was performed at 25 °C for 0, 5, 12, 21, and 30 days in separate 500 ml sealed glass bottles (filled with medium and 20 g of sediment sample). The bottle was shaken twice a day and kept sealed during the incubation. The anaerobic enrichment cultures were diluted with 9 ml of sterile seawater and spread onto marine agar 2216 (MA; BD) after different periods of incubation (0, 5, 12, 21, and 30 days). The plates were then incubated at 28 °C for 7-10 days. Strain colonies were selected from cultures enriched for different periods based on their colony size, colour, and shape and were then restreaked on fresh MA plates to purity and stored at -80 °C in 20% (v/v) glycerol.

Identification of each isolate was performed by PCR amplification of the almost full-length 16S rRNA gene, using 27F (5′-AGAGTTTGATCCTGGCTCAG-3′) and 1492R (5′-GGTTACCTTGTTACGACTT-3′) primers, and subsequent sequencing [1]. Amplification products were purified using a PCR product purification kit (Tiangen) and then ligated into the vector pGM-T (Tiangen). Universal primers T7 and SP6 were used for sequencing, which was performed by Shanghai Sunny Biotechnology (China). A nearly complete 16S rRNA gene sequence (1461 bp) was obtained and submitted to the GenBank/EMBL/DDBJ databases; preliminary screening for similarity was carried out with the BLAST algorithm.

Based on the sequence similarity through the NCBI BLAST search with type species, 86% could be used as an order-level cut-off [2], 86%-90% could be approximately identified as a candidate novel family [3], 90%-95% could be approximately identified as a candidate novel genus [3], and 98% was used as a species-level cut-off [4].

A phylogenetic trees was reconstructed using mega 7.0 software package: maximum-likelihood. Sequences were aligned using Clustalw in the mega 7.0 software package.The node support of the tree topology was evaluated using bootstrapping estimation of 1000 replicates for the method. The best substitution model (K2+G+I) for maximum-likelihood was determined under the lowest BIC selection scores (Bayesian Information Criterion). The detailed display and annotation of phylogenetic tree were made by iTOLs (http://itol.embl.de/), which is a web-based tool for the display, manipulation and annotation of phylogenetic trees.

**Microbial community composition based on tag-encoded amplicon sequence analysis**

The sequences were analyzed using the Quantitative Insights into Microbial Ecology (QIIME, version 1.9.0) software package [5] with the default parameters. Chimeras were identified and filtered using UPARSE with the UCHIME algorithm and the ChimeraSlayer reference database [6], which is considered to be fast and sensitive [7].

Next, 16S rDNA tag sequences were assigned to operational taxonomic units (OTUs, 97% sequence identity) using RDP 10 [8]. Beta diversity was calculated on normalized OTU tables using weighted UniFrac [9]. We also analysed beta diversity using Bray-Curtis similarity, which does not consider the phylogenetic information of the OTUs when calculating similarity, and these results were consistent with the UniFrac results. After the data were determined to meet the relevant assumptions, we used multiple one-way ANOVAs to determine significant changes in diversity during each period of enrichment culturing.

**Sequence quality control, Gene prediction, taxonomy, and functional annotation for metatranscriptomic sequencing**

Next, 3’ and 5’ ends were stripped using SeqPrep (https://github.com/jstjohn/SeqPrep). Low-quality reads (having a length<50 bp, a quality value <20, or N bases) were removed by Sickle (<https://github.com/najoshi/sickle>). Ribosomal RNA reads were removed *in silico* using the Kmer-based tool DUK [10], and Open reading frames (ORFs) from each sample were predicted using TransGeneScan (http://sourceforge.net/projects/transgenescan/). All sequences with a 95% sequence identity (90% coverage) were clustered into a non-redundant gene catalogue by CD-HIT (http://www.bioinformatics.org/cd-hit/).

The reads that remained after quality control were mapped to representative genes with 95% identity, and the Fragments Per Kilobase of transcript per Million fragments mapped (FPKM) were evaluated using RSEM (http://deweylab.biostat.wisc.edu/rsem/).

BLASTP (Version 2.2.28+, <http://blast.ncbi.nlm.nih.gov/Blast.cgi>) was employed for taxonomic annotations by aligning non-redundant gene catalogues against the NCBI NR database with an E-value cutoff of 1e-5. GO annotation was performed using Blast2GO (http://www.blast2go.com/b2ghome), aligning sequences to the GO database (Gene Ontology, <http://www.geneontology.org).> Clusters of Orthologous Group (COG) annotation was performed using BLASTP against the eggNOG database (V4.5) (evolutionary genealogy of genes: Non-supervised Orthologous Groups, http://eggnog.embl.de/). KEGG annotation was performed using BLASTP against KEGG database (Kyoto Encyclopedia of Genes and Genomes, http://www.genome.jp/kegg/). All BLAST E-value cutoffs were 1e-5.

**Microbial Community Analysis by almost full-length 16S rRNA and PacBio SMRT Sequencing after Targeted Culture**

The medium for target bacterial culture was marine agar 2216 (MA; BD) with 5 Mm NaNO_3_ and vitamin B complex (1 × vitamin solution and 1 × B_12_ solution(0.1 %)) [11]. Culture media and all other materials that were used for culturing were placed in the anaerobic cabinet 24 h before use to reduce to anaerobic conditions. All processing and culturing took place under anaerobic conditions in a ELECTROTEK AW400GS workstation at 28 °C for 7-10 days.

Then we washed all the isolates which grew on the plates with 1×PBS buffer. Total DNA was extracted by using QIAamp DNA Mini Kit (Qiagen) and further purification using a Power Clean ProDNA Clean-up Kit from MoBio for DNA. The quality of the extracted DNA was checked by agarose gel electrophoresis on a 0.8% gel and spectrophotometry (optical density at 260 nm/280 nm ratio, Thermo Fisher Scientific, Waltham, MA). All DNA samples were stored at −20 °C for further experiments.

The forward 27F (5′-AGAGTTTGATCCTGGCTCAG-3′) and the reverse 1492R (5′-GGTTACCTTGTTACGACTT-3′) primers were used to amplify the full-length 16S rRNA gene with the PCRBIO Taq DNA polymerase (PCR Biosystems Ltd., London, UK). The primers contained a set of 16-nucleotide barcodes. The volume of the final reaction mixture was 50 µL. Each sample contained 1 × PCRBIO reaction buffer, 10 ng of template DNA, 10 pmol of each primer, and 1.0 U of Taq DNA polymerase. The reaction conditions were as follows: 95 °C for 5 min, then 30 cycles at 95 °C for 30 s, 58 °C for 45 s, and 72 °C for 1 min with a final extension of 72 °C for 7 min [12]. The Agilent DNA 1000 Kit and an Agilent 2100 Bioanalyser (Agilent Technologies, Santa Clara, CA) were used to check the quality of the PCR products. Purified PCR products were used to construct DNA libraries with the Pacific Biosciences Template Prep Kit 2.0 (Pacific Biosciences). The P6-C4 chemistry on the PacBio RS II platform was used to sequence the purified amplification products.

In order to decrease the sequencing error rate, Pacbio circular consensus sequencing (CCS) reads were derived from the multiple alignments of sub-reads. In CCS, the DNA polymerase reads a ligated circular DNA template multiple times, which can effectively generate a consensus sequence from multiple reads of a single molecule[13]

Raw data were generated under the protocol RS_ ReadsOfinsert.1. Sequencing adapters and low-quality sequences were then filtered out using the software package of Mothur version (v.1.30.1 [14]). UCHIME is used for detection and removal of chimeric sequences with two or more segments [7]. The high-quality reads were aligned in accordance with Silva alignment protocols and clustered into OTUs.

**Genome sequencing and assembly for the isolates in this study**

Genomic DNA ot the isolates were extracted using MiniBEST Bacteria Genomic DNA Extraction Kit (TaKaRa, China). The harvested DNA was detected by the agarose gel electrophoresis and quantified by Qubit. Whole-genome sequencing was performed on the Illumina HiSeq PE150 platform. A-tailed, ligated to paired-end adaptors and PCR amplified with a 350 bp insert was used for the library construction at the Beijing Novogene Bioinformatics Technology Co., Ltd. Illumina PCR adapter reads and low quality reads from the paired-end were filtered by the step of quality control using our own compling pipeline. All good quality paired reads were assembled using the SOAP denovo (http://soap.genomics.org.cn/soapdenovo.html) into a number of scaffolds. Then the filter reads were handled by the next step of the gap-closing.

**
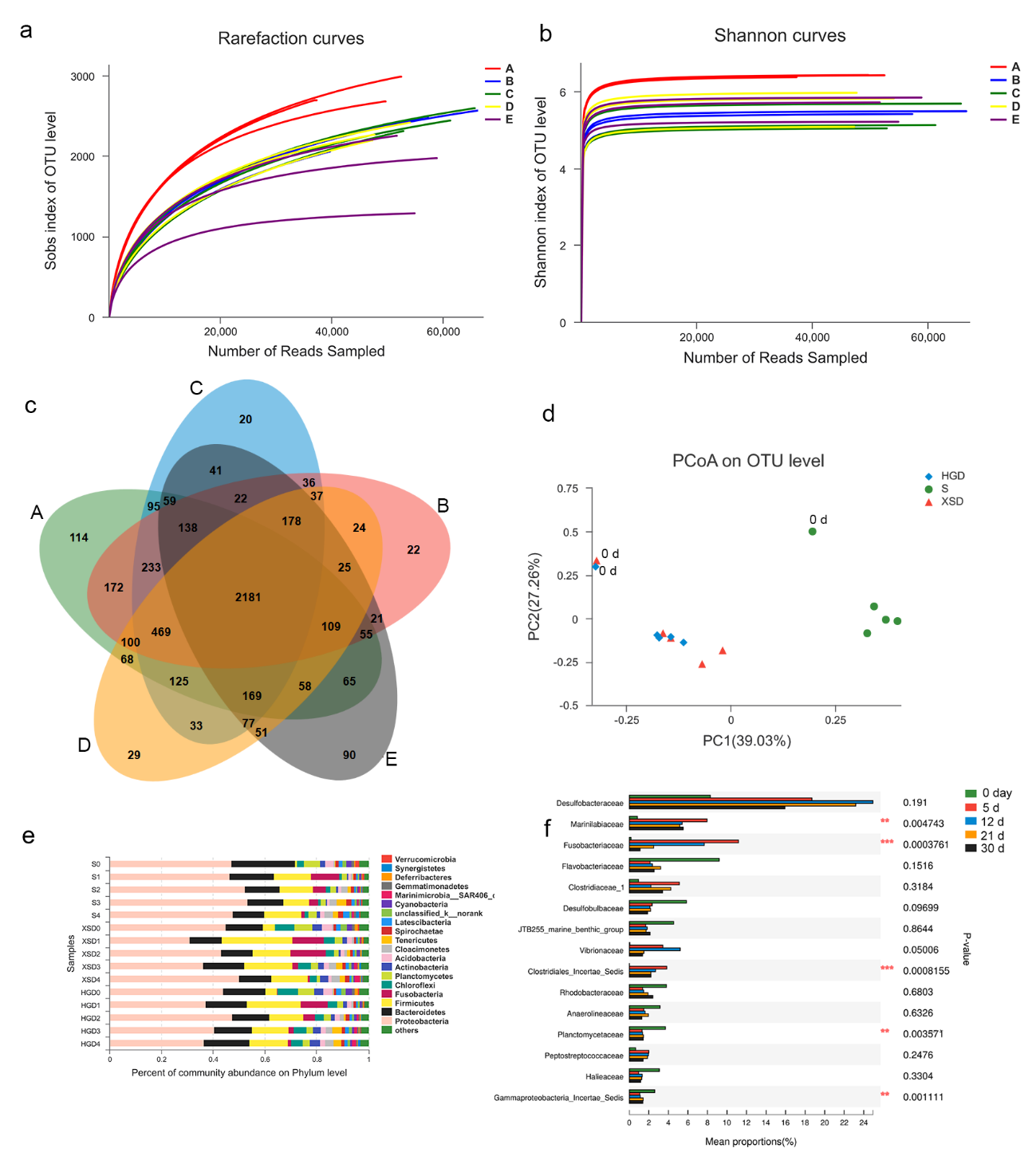
**

**Supplementary Figure 1**. **Species richness and diversity of bacterial phylotypes observed in different stages of enrichment culturing of three sediment samples.**

**(a)** Rarefaction curves of the 3 sediments during five periods of enriching by OUT analysis. The letters A-E indicate 0, 5, 12, 21, and 30 d of enrichment culturing. **(b)** Shannon diversity index curves of the 3 sediments during five periods of enriching by OUT analysis. The letters A-E indicate 0, 5, 12, 21, and 30 d of enrichment culturing. **(c)** Venn diagram of 16S OTUs during five periods of enriching the three sediments. The letters A-E indicate 0, 5, 12, 21, and 30 d of enrichment culturing. **(d)** PCoA of microbial communities abundance based on weighted UniFrac metrics from five stages of enrichment culturing. Different samples are indicated by different colours. **(e)** Relative abundances (%) of dominant lineages (phylum level) at different stages of the enrichment culture. **(f)** Relative abundances of significantly different families in cultures enriched for 0, 5, 12, 21, and 30 d. All shown phyla are from the 15 most abundant microorganisms. The one-way ANOVA test was used to evaluate the significance of differences between indicated groups. n=3, **P<0.01, ***P<0.001.


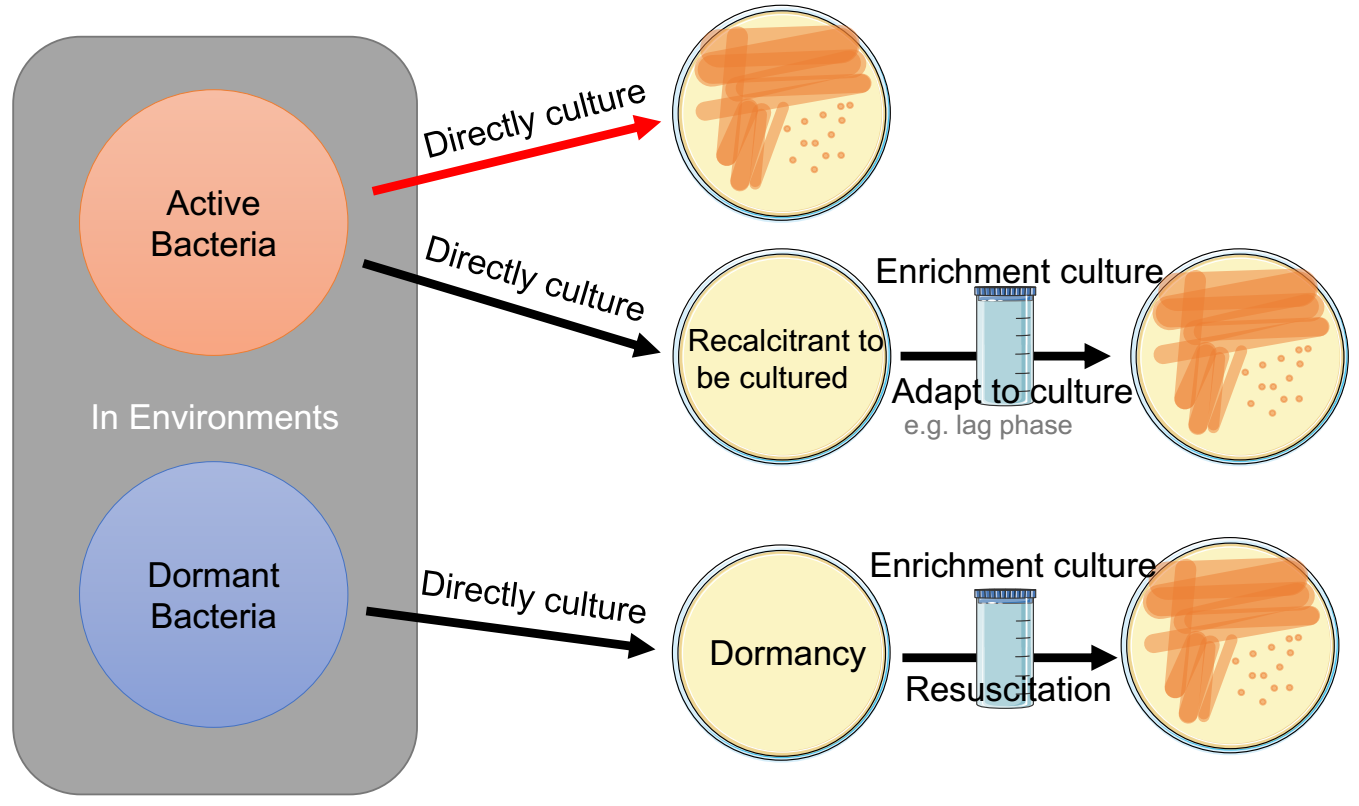


**Supplementary Figure 2. Schematic diagram of bacterial culturable mechanism during the enrichment culture.** Generally, bacteria in natural environments have two states, one is active state and the other is dormancy[15]. We hypothesized that the enrichment-culture in this study help some active species adapt and grow on the MA medium, like the bacteria live through the lag phase. Meanwhile, enrichment-culture could aid the resuscitation of dormant or viable but nonculturable bacteria, then help bacteria be cultured on MA medium.





**Supplementary Figure 3. Enrichment culture increases the percent of dormant taxa and decreases active diversity.** Percent of active taxa in enrichment culturing, as determined by 16S rRNA: 16S rRNA gene (n=3, error bars are s.e.m.).


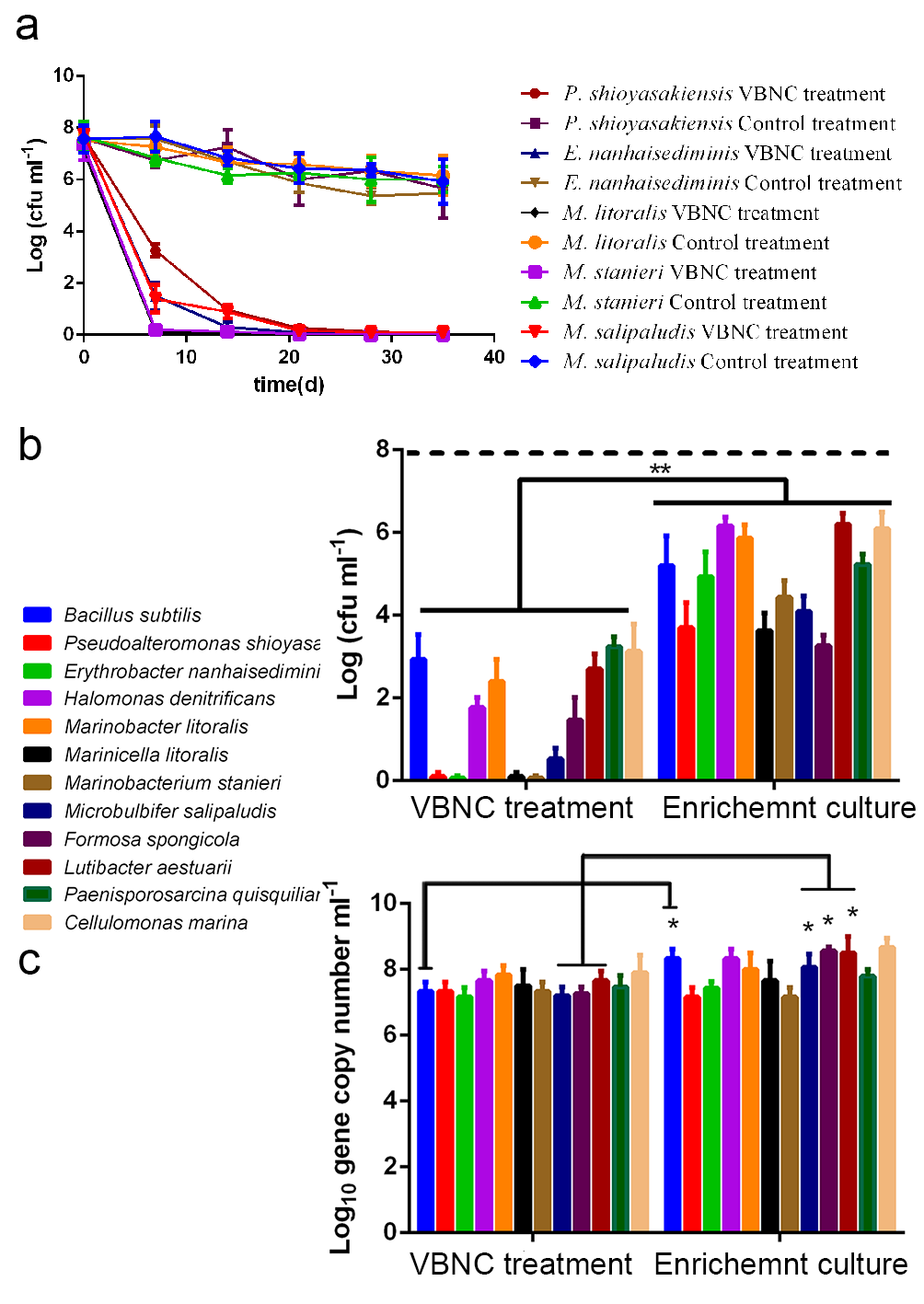


**Supplementary Figure 4. Strains transition to, and resuscitation from VBNC states (a)** The growth status of five of ten species receiving the VBNC treatment for 0 to 35 days is shown. Most of the five strains could be induced into the VBNC state within 2 weeks. *P. shioyasakiensis* stands for *Pseudoalteromonas shioyasakiensis*, *E. nanhaisediminis* for *Erythrobacter nanhaisediminis*, *M. litoralis* for *Marinicella litoralis*, *M. stanieri for Marinobacterium stanieri*, and *M. salipaludis* for *Microbulbifer salipaludis*. VBNC treatment indicates that strains were washed with seawater and then incubated for 0, 5, 10, 15, 20, 25, 30 and 35 days at 4 °C in seawater. Control treatment indicates that strains were incubated for 0, 5, 10, 15, 20, 25, 30 and 35 days at 28 °C in seawater (Mean ± s.e., n=3 biological replicates for each species tested). **(c)** Resuscitation of VBNC cells using the enrichment culture method. The term VBNC treatment indicates that VBNC cells were spread onto MA medium and incubated at 28 °C for 6 days. The term enrichment culture indicates that VBNC cells were incubated in enrichment culture medium at 25 °C for 5 days, spread onto MA medium and then incubated at 28 °C for 1 day. Culturable cells were enumerated based on the formation of visible colonies on MA medium. *Bacillus subtilis*, which was isolated in all five stages, was selected as a control strain. The other nine strains were isolated only after the enrichment culture treatment. The dotted line represents the initial number of CFUs of each strain (before VBNC treatment) (**P<0.001, n=3). **(d)** Number of copies of the 16S rRNA gene from different bacteria receiving the different treatments. The term VBNC treatment indicates the number of copies of the 16S rRNA gene of VBNC cells in seawater, and the term enrichment culture indicates the number of copies of the 16S rRNA gene of VBNC cells that had been incubated in enrichment culture medium for 5 days. (Mean ± s.d., * indicates P<0.05, n=3 biological replicates for each species tested).


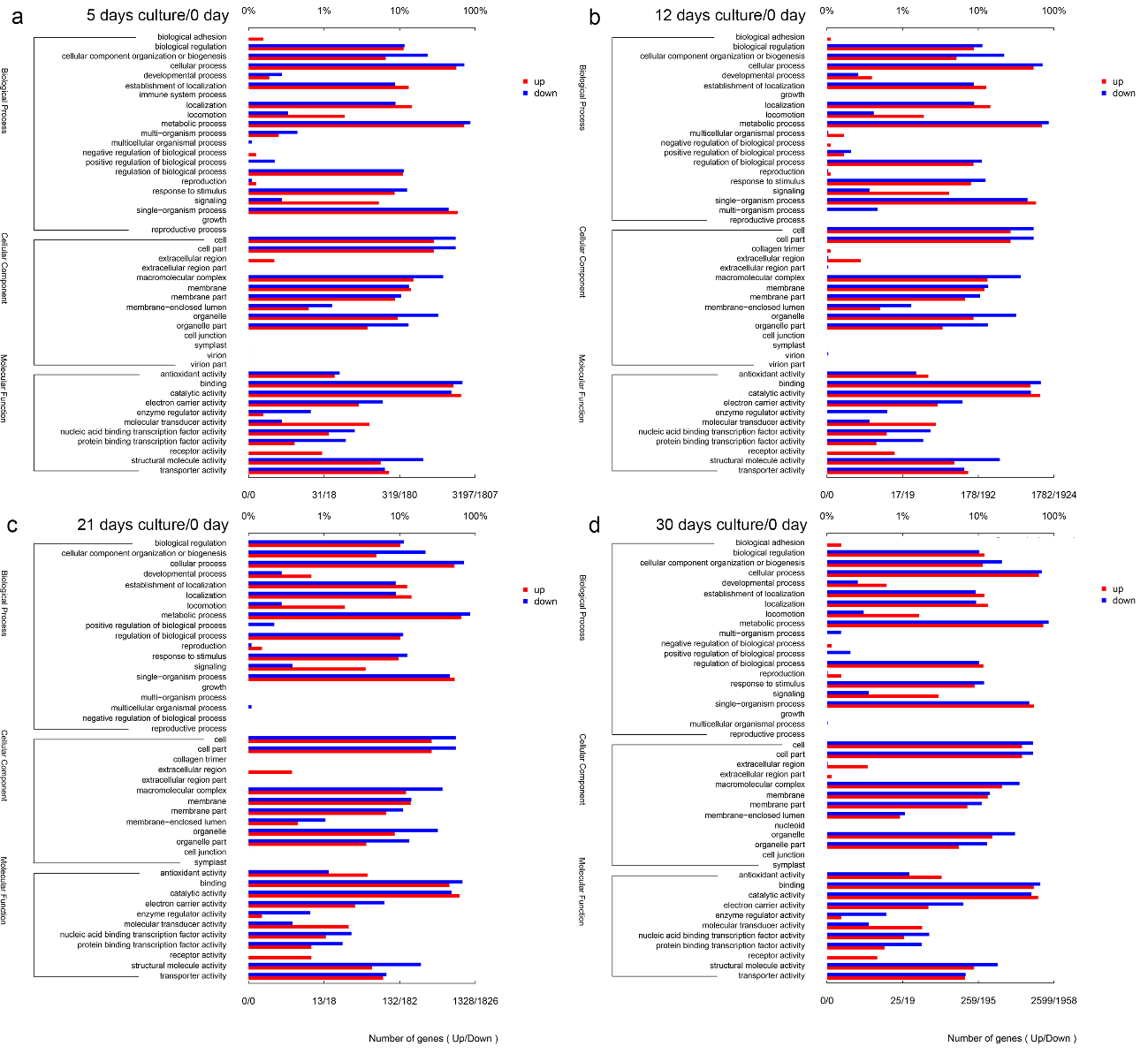


**Supplementary Figure 5. Comparative transcriptional activities of the three sediment sample communities under enrichment treatment. (a)** Functional assignment of transcripts based on GO categories after 5 days of enrichment culturing. **(b)** Comparing functional assignment of transcripts based on GO categories from samples enriched for 12 days and control samples. **(c)** Comparing functional assignment of transcripts based on GO categories from samples enriched for 21 days and control samples. **(d)** Comparing functional assignment of transcripts based on GO categories from samples enriched for 30 days and control samples.


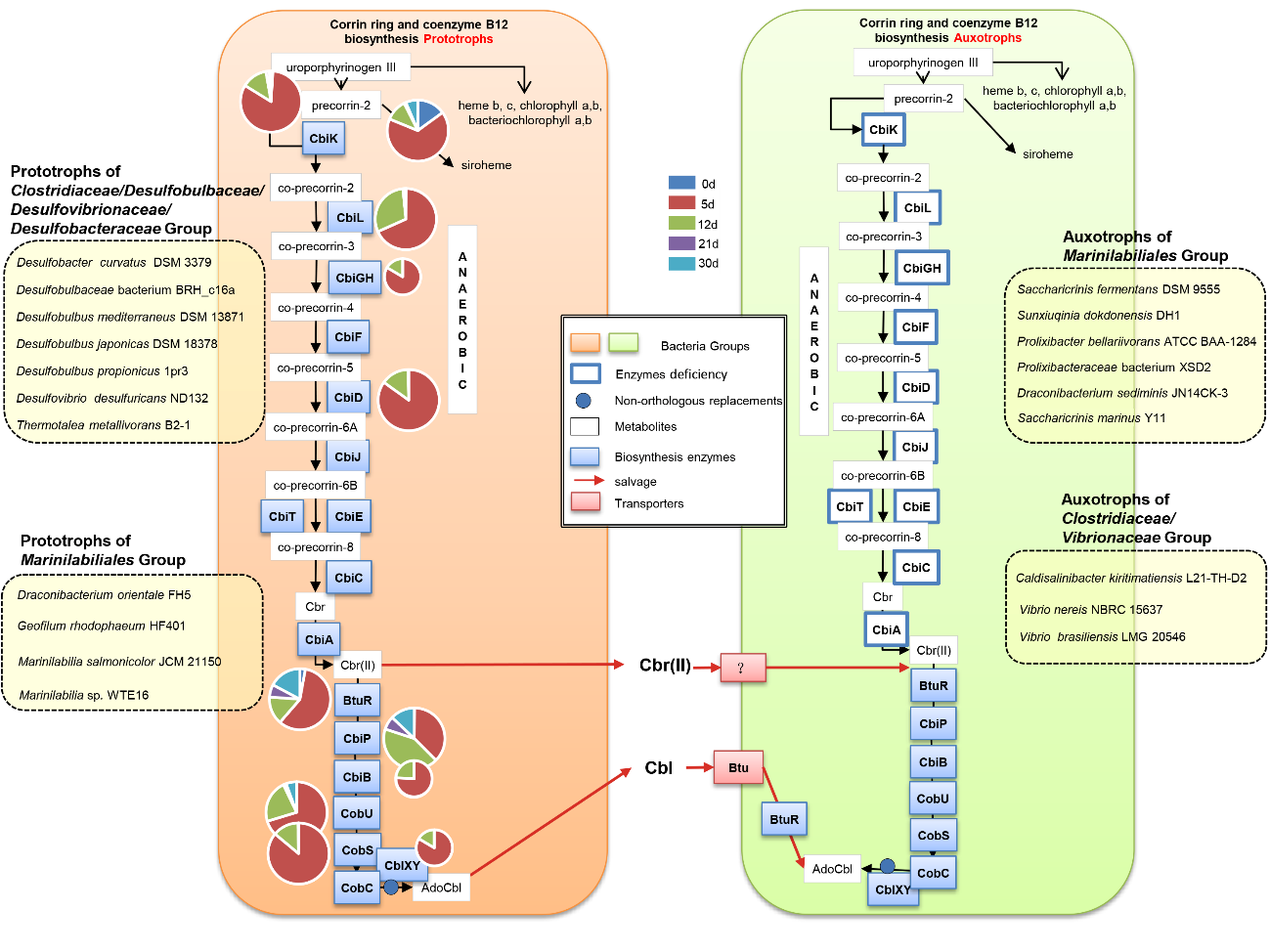


**Supplementary Figure 6. Overview of Cobalamin biosynthesis.** A portion of Cobalamin biosynthetic pathway that includes metabolites were shown in white rectangles: Cbr(II), cob(II)yrinate a,c-diamide; Cbr(I), cob(I)yrinate a,c-diamide; Biosynthetic enzymes: CbiX, sirohydrochlorin cobaltochelatase; CbiL, cobalt-precorrin-2 C20-methyltransferase; CbiG-CbiH, bifunctional cobalt-precorrin-5A hydrolase / cobalt-factor-3 methyltransferase; CbiF, cobalt-precorrin-4 (C11)-methyltransferase; CbiD, cobalt-precorrin-5B (C1)-methyltransferase; CbiJ, cobalt-precorrin-6A reductase; CbiE, bifunctional cobalt-precorrin-7 (C5)-methyltransferase / cobalt-precorrin-6B (C15)-methyltransferase (decarboxylating); CbiT, cobalt-precorrin-6B (C15)-methyltransferase (decarboxylating); CbiC, cobalt-precorrin-8 methylmutase; CbiA, cobyrinate a,c-diamide synthase; Btu refers to the ABC-type corrinoid transporter and BtuB TonB-dependent outer membrane corrinoid receptor which is found in most members. Enzymes involved biosynthesis appear in blue rectangles and those in salvage in light red rectangles: CobB, glutamine-hydrolysing hydrogenobyrinic acid a,c-diamide synthase; CobNST and CobN-ChlD, alternative cobaltochelatase complexes; BtuR, alternative corrinoid adenosyltransferases; CbiP, cobyric acid synthase; CobU, bifunctional adenosylcobinamide kinase / adenosylcobinamide-phosphate guanylyltransferase; CobS, adenosylcobinamide-GDP ribazoletransferase; CobC and CblXY, alternative alpha-ribazole-5‘-phosphate phosphatases. Non-orthologous replacements are indicated by filled blue circles. Red arrows indicate pathways for recycling and salvage of adenosylcobalamin precursors. Common alternative enzyme names appear next to blue rectangles. Note that this figure includes corrections to the last steps of biosynthesis that have been previously reported but infrequently reflected in recent publications. Auxotroph groups, which mainly contain *Marinilabiliales* bacteria, indicate the microbes are unable to synthesize the Cobalamin *de novo*, and missing most of the pathway genes. Phototroph groups, which mainly contain *Vibrionaceae*, *Clostridiaceae*, *Desulfobulbaceae*, *Desulfovibrionaceae*, and *Desulfobacteraceae* bacteria, indicate that the microbes are able to synthesize the Cobalamin *de novo*. *Desulfobacteraceae* node have no correlations with *Prolixibacteraceae* node and *Marinilabiliaceae* node, however, the bacteria within this family were high enriched and became the dominant family during the enrichment culture, as a result, we also analyzed the genome of this family members. Pie Chart indicates the total Fragments Per Kilobase of transcript per Million fragments mapped (FPKM) of the corresponding gene in each sediment sample.

**Supplementary references**

1. Wang ZJ, Liu QQ, Zhao LH, Du ZJ, Chen GJ: **Bradymonas sediminis gen. nov., sp. nov., isolated from coastal sediment, and description of Bradymonadaceae fam. nov. and Bradymonadales ord. nov**. *Int J Syst Evol Microbiol* 2015, **65**(Pt 5):1542-1549.

2. Munoz R, Rossello-Mora R, Amann R: **Revised phylogeny of Bacteroidetes and proposal of sixteen new taxa and two new combinations including Rhodothermaeota phyl. nov**. *Systematic and applied microbiology* 2016, **39**(5):281-296.

3. Browne HP, Forster SC, Anonye BO, Kumar N, Neville BA, Stares MD, Goulding D, Lawley TD: **Culturing of ‘unculturable’ human microbiota reveals novel taxa and extensive sporulation**. *Nature* 2016, **533**(7604):543-546.

4. Kim M, Oh HS, Park SC, Chun J: **Towards a taxonomic coherence between average nucleotide identity and 16S rRNA gene sequence similarity for species demarcation of prokaryotes**. *International journal of systematic and evolutionary microbiology* 2014, **64**(Pt 2):346-351.

5. Caporaso JG, Kuczynski J, Stombaugh J, Bittinger K, Bushman FD, Costello EK, Fierer N, Pena AG, Goodrich JK, Gordon JI *et al*: **QIIME allows analysis of high-throughput community sequencing data**. *Nat Methods* 2010, **7**(5):335-336.

6. Haas BJ, Gevers D, Earl AM, Feldgarden M, Ward DV, Giannoukos G, Ciulla D, Tabbaa D, Highlander SK, Sodergren E *et al*: **Chimeric 16S rRNA sequence formation and detection in Sanger and 454-pyrosequenced PCR amplicons**. *Genome Res* 2011, **21**(3):494-504.

7. Edgar RC, Haas BJ, Clemente JC, Quince C, Knight R: **UCHIME improves sensitivity and speed of chimera detection**. *Bioinformatics* 2011, **27**(16):2194-2200.

8. Cole JR, Wang Q, Cardenas E, Fish J, Chai B, Farris RJ, Kulam-Syed-Mohideen AS, McGarrell DM, Marsh T, Garrity GM *et al*: **The Ribosomal Database Project: improved alignments and new tools for rRNA analysis**. *Nucleic Acids Res* 2009, **37**(Database issue):D141-145.

9. Lozupone C, Knight R: **UniFrac: a new phylogenetic method for comparing microbial communities**. *Appl Environ Microbiol* 2005, **71**(12):8228-8235.

10. Beam JP, Jay ZJ, Schmid MC, Rusch DB, Romine MF, Jennings Rde M, Kozubal MA, Tringe SG, Wagner M, Inskeep WP: **Ecophysiology of an uncultivated lineage of Aigarchaeota from an oxic, hot spring filamentous 'streamer' community**. *ISME J* 2016, **10**(1):210-224.

11. Flood BE, Jones DS, Bailey JV: **Sedimenticola thiotaurini sp. nov., a sulfur-oxidizing bacterium isolated from salt marsh sediments, and emended descriptions of the genus Sedimenticola and Sedimenticola selenatireducens**. *Int J Syst Evol Microbiol* 2015, **65**(8):2522-2530.

12. Jin H, Mo LX, Pan L, Hou QC, Li CJ, Darima I, Yu J: **Using PacBio sequencing to investigate the bacterial microbiota of traditional Buryatian cottage cheese and comparison with Italian and Kazakhstan artisanal cheeses**. *J Dairy Sci* 2018, **101**(8):6885-6896.

13. Wagner J, Coupland P, Browne HP, Lawley TD, Francis SC, Parkhill J: **Evaluation of PacBio sequencing for full-length bacterial 16S rRNA gene classification**. *Bmc Microbiology* 2016, **16**.

14. Schloss PD, Westcott SL, Ryabin T, Hall JR, Hartmann M, Hollister EB, Lesniewski RA, Oakley BB, Parks DH, Robinson CJ *et al*: **Introducing mothur: open-source, platform-independent, community-supported software for describing and comparing microbial communities**. *Appl Environ Microbiol* 2009, **75**(23):7537-7541.

15. Jones SE, Lennon JT: **Dormancy contributes to the maintenance of microbial diversity**. *Proceedings of the National Academy of Sciences of the United States of America* 2010, **107**(13):5881-5886.
